# Silk-Elastin-like Polymers for Acute Intraparenchymal Treatment of the Traumatically Injured Spinal Cord: A First Systematic Experimental Approach

**DOI:** 10.3390/pharmaceutics14122713

**Published:** 2022-12-03

**Authors:** Pau González, Carlos González-Fernández, Alfredo Maqueda, Virginia Pérez, Sara Escalera-Anzola, Ángel Rodríguez de Lope, Francisco Javier Arias, Alessandra Girotti, Francisco Javier Rodríguez

**Affiliations:** 1 Laboratory of Molecular Neurology, Hospital Nacional de Parapléjicos, 45071 Toledo, Spain; 2 Smart Devices for NanoMedicine Group University of Valladolid, 47003 Valladolid, Spain; 3 Unidad de Excelencia Instituto de Biomedicina y Genética Molecular (IBGM), Universidad de Valladolid and Consejo Superior de Investigaciones Científicas (CSIC), 47003 Valladolid, Spain; 4 Department of Neurosurgery, Hospital Universitario de Toledo, 45007 Toledo, Spain

**Keywords:** spinal cord injury, silk-elastin-like polymers, (EIS)_2_-RGD6

## Abstract

Despite the promising potential of hydrogel-based therapeutic approaches for spinal cord injury (SCI), the need for new biomaterials to design effective strategies for SCI treatment and the outstanding properties of silk-elastin-like polymers (SELP), the potential use of SELPs in SCI is currently unknown. In this context, we assessed the effects elicited by the in vivo acute intraparenchymal injection of an SELP named (EIS)_2_-RGD6 in a clinically relevant model of SCI. After optimization of the injection system, the distribution, structure, biodegradability, and cell infiltration capacity of (EIS)_2_-RGD6 were assessed. Finally, the effects exerted by the (EIS)_2_-RGD6 injection—in terms of motor function, myelin preservation, astroglial and microglia/macrophage reactivity, and fibrosis—were evaluated. We found that (EIS)_2_-RGD6 can be acutely injected in the lesioned spinal cord without inducing further damage, showing a widespread distribution covering all lesioned areas with a single injection and facilitating the formation of a slow-degrading porous scaffold at the lesion site that allows for the infiltration and/or proliferation of endogenous cells with no signs of collapse and without inducing further microglial and astroglial reactivity, as well as even reducing SCI-associated fibrosis. Altogether, these observations suggest that (EIS)_2_-RGD6—and, by extension, SELPs—could be promising polymers for the design of therapeutic strategies for SCI treatment.

## 1. Introduction

Spinal cord injury (SCI) is a devastating neuropathological condition that induces major long-term functional disabilities, thereby leading to huge physical, psychological, social, and economic burden for patients and their families. Unfortunately, despite the significant advances in its early medical and surgical management, as well as our understanding of its pathophysiology, there is no currently accepted treatment for SCI [1]. From a neuropathological point of view, SCI is generally characterized by two consecutive phases. The primary injury phase comprises axonal damage, neuronal and glial cell loss, and vascular disruption, which take place due to mechanical spinal cord trauma. Subsequently, the secondary injury phase is initiated, which is characterized by a wide range of complex and inter-related cellular and molecular processes, usually leading to damage to the initially non-affected neural cells and circuits that surround the primary injury core. Ultimately, the progression of the injury results in the formation of a cystic cavity filled with fluid, macrophages, and fibroblasts surrounded by a dense glial scar that, together with the limited regenerative capacity of the lesioned spinal cord, greatly hinder endogenous tissue regeneration [2,3].

Consequently, the use of biomaterials in SCI has gained great attention from the scientific community due to their promising potential capacity to induce the replacement of the lost spinal cord tissue with a new pro-regenerative one and/or to act as a permissive bridge favouring axonal regeneration across the lesioned areas, ultimately improving functional recovery [4,5]. Moreover, due to the complex and multi-faceted nature of SCI, it has been pointed out that a combination of different therapeutic approaches will be needed to synergistically overcome the multiple deleterious aspects that limit spinal cord regeneration. In this regard, promising results have been obtained through the combination of biomaterials with different cells or drugs, as such a combination will be able to circumvent many of the classical handicaps of single-cell transplantation or drug delivery systems [6,7,8]. Despite the existence of slight discrepancies, there is now a consensus on the characteristics that should ideally be displayed by those biomaterials to be used in the lesioned spinal cord: (1) porosity—the biomaterial should have enough porosity to allow for cell infiltration and diffusion of soluble factors such as nutrients, ions, and waste products; (2) injectability—biomaterials should be injectable to allow for minimally invasive delivery; (3) in situ polymerization—the biomaterial should polymerize in the injection site to conveniently fill the entire spinal cord lesions, which usually display irregular geometries; (4) biodegradability—the biomaterial should be biodegradable, in order to avoid the need for surgical removal of the implant; (5) biocompatibility and immunogenicity—the biomaterial, its degradation products, and any potential component needed for its proper polymerization should be non-toxic and non-immunogenic; (6) mechanical properties—the biomaterial should have similar mechanical properties to those observed in the spinal cord; (7) tailorability—the biomaterial should be easily modifiable, to change its physicochemical properties and/or incorporate different bio-active motifs; (8) combinability—the biomaterials should be combinable with potential therapeutic drugs and/or cells; and (9) the biomaterial should be able to be easily manufactured, purified, and sterilized without batch-to-batch variability, while avoiding the use of aggressive protocols that could lead to a reduction in the viability or bioactivity of cells or therapeutic compounds that could be combined with it [5,6,9,10,11,12].

Hydrogels, which are defined as cross-linked hydrophilic polymers with high water content, are promising candidates for the development of effective biomaterial-based therapeutic approaches for SCI, as they can fulfil many of the aforementioned requirements [9,13,14,15,16,17]. In particular, an increasing number of studies have focused on the generation of hydrogels using self-assembling naturally derived peptides, as they can combine the advantages of natural hydrogels with extremely high design flexibility and control over the polymer composition and properties, and they also lack the batch-to-batch variability displayed by synthetic ones [14,18,19]. Among them, elastin-like polypeptides (ELPs) have been shown to possess a versatile and ample range of desirable characteristics. Briefly, the basic structure of ELPs is a repeating sequence (VPGXG, where X represents any natural or modified amino acid, except for L-proline) having its origin in the repeating sequences found in mammalian elastin. One of the most striking properties of ELPs is that they can be injected and, subsequently, polymerize in situ, due to the fact that they exhibit a reversible phase transitional behaviour in response to changes in temperature (they only polymerize above a certain transition temperature, (Tt), while below Tt the polymer chains remain disordered). Moreover, the self-assembly of ELPs is based on the occurrence of physical interactions due to temperature changes and, therefore, does not require the addition of potentially toxic compounds or aggressive protocols to induce polymer cross-linking, allowing their combination with potential therapeutic cells or drugs without inducing cell damage or reducing drug bioactivity. In addition, ELPs can be produced by genetic engineering techniques, leading to outstanding flexibility in their design, almost total control over their composition and properties, and extremely low batch-to-batch variability. In this line, the physicochemical properties of ELPs can be efficiently modified, and different bioactive motifs can be easily incorporated in their composition, in order to improve their functionality. Furthermore, as elastin is a naturally present protein in mammals and its degradation products are simple amino acids, they usually exhibit high biocompatibility and a lack of immunogenicity. Additionally, they are typically degradable and can be used to generate different structures such as particles, fibres, sheets, and porous hydrogel scaffolds for tissue engineering purposes. Finally, it should be noted that ELPs retain most of the mechanical properties of natural elastin and, consequently, display a great elasticity and resistance to fatigue [20,21,22,23,24,25,26,27].

Besides the encouraging characteristics of ELPs, it also should be noted that several studies have evaluated various specific aspects related to their potential use in the central nervous system (CNS) [28]; for instance, it has been reported that functionalized ELPs were able to induce cell adhesion and neurite outgrowth in PC12 cell cultures without affecting cell survival [29,30,31,32]. Moreover, they can be effectively used as drug delivery platforms in the CNS [33,34,35,36]. Furthermore, they can be used to encapsulate different cell types that display an evident therapeutic potential for the treatment of traumatic CNS injuries, such as neural progenitor cells, induced pluripotent stem cells, oligodendrocyte precursor cells, and mesenchymal cells, without affecting their viability [37,38,39,40,41,42]. In addition, it has been shown that incubation of microglial and astroglial cell lines with ELPs did not influence cell survival [43], and their incubation with cultured primary astroglial cells did not induce an inflammatory response [44]. Finally, it has been demonstrated that the administration of ELPs in a model of intracerebral haemorrhage was able to reduce hematoma volume as well as to reduce the injury-associated vascular leakage and microglia/macrophage reactivity [45].

Altogether, the previously detailed observations clearly indicate that ELPs exhibit a wide range of physicochemical and biological properties that make them highly suitable for use in SCI. However, one of the major drawbacks of physical ELP-based hydrogels is the relatively weak nature of the physical interaction mediated by the ELP hydrophobic domains, leading to inadequate stability for robust and long-term hydrogel maintenance during tissue regeneration [22,23,27,46]. To overcome this problem, and given the good performance of silk fibroin-based biomaterials in different CNS pathologies, including SCI [47], silk fibroin domains have been incorporated into the ELP backbone, giving rise to the so-called silk-ELPs (SELPs), which retain the abovementioned physicochemical features of ELPs while presenting better mechanical properties, due to the stability and irreversibility provided by the incorporation of silk motifs [22,23,27,46].

Taking into account all of these findings, together with the need for new biomaterials to develop effective treatments for SCI, it is somewhat surprising that there is no currently available information on the potential use of SELPs in this neuropathological condition. Consequently—and as a first experimental approach aiming to evaluate the potential use of SELPs in SCI—in this study, we evaluate the effects elicited by the minimally invasive acute intraparenchymal injection of an SELP-based injectable corecombinamer named (EIS)_2_-RGD6 in an in vivo clinically relevant rat model of contusive SCI [2,48]. As has been previously reported [49,50,51,52], this SELP spontaneously and rapidly forms a physical porous nanofibrillar hydrogel under physiological conditions, allows for cellular adhesion and proliferation, displays good cytocompatibility and long-term stability, can be used as a drug- and cell-delivery platform, and does not induce an inflammatory response when subcutaneously injected. Briefly, after determination of the best injection system, the in vivo distribution, structure, porosity, biodegradability, and cell infiltration capacity of (EIS)_2_-RGD6 were assessed. Finally, the effects exerted by (EIS)_2_-RGD6 in SCI-associated motor functional recovery, myelin preservation, astroglial and microglia/macrophage reactivity, and fibrosis were evaluated.

## 2. Materials and Methods

### 2.1. (EIS)_2_-RGD6 Design, Biosynthesis, and Purification

As previously stated, for the present study we selected the SELP named (EIS)_2_-RGD6, which has been designed to comprise a hydrophilic block {E block: [(VPGVG)_2_-(VPGEG)-(VPGVG)_2_]_10_}, a hydrophobic block [I block: (VGIPG)_60_] and a silk-like block {S block: [V(GAGAGS)_5_G]_2_}, which were functionalized through the inclusion of six RGD cell adhesion sequences [(AVTGRGDSPASS)_6_], resulting in the following abbreviated amino acid sequence: MESLLP-{[(VPGVG)_2_-VPGEG-(VPGVG)_2_]_10_-(VGIPG)_60_-[V(GAGAGSG)_5_]_2_G}-[(VPGIG)_5_-AVTGRGDSPASS]_6_ [49,50]. The biosynthesis, characterization, production, purification, and gelation properties of (EIS)_2_-RGD6 have been previously described [49,50,51,52]. Briefly, the genetic construction of (EIS)_2_-RGD6 was carried out using standard gene-engineering and molecular biology methods [49,50,51]. Moreover, (EIS)_2_-RGD6 recombinant production was performed in *Escherichia coli* BLR(DE3) (Invitrogen, Waltham, MA, USA), as previously detailed [49,50,51,52]. Subsequently, (EIS)_2_-RGD6 purification was carried out using several cooling and heating purification cycles (Inverse Temperature Cycling), taking advantage of the ability of (EIS)_2_-RGD6 to precipitate above its Tt (approximately 16.8 °C in PBS), and endotoxins were removed by performing additional NaCl (NAC02, Formedium, Norfolk, UK) and NaOH (S/4880760, Fischer Scientific, Madrid, Spain) treatments [49,50,51,52]. Finally, (EIS)_2_-RGD6 was dialyzed against ultra-pure water, sterilized by filtration of the solution through 0.22 µm filters (FB12566502, Fischer Scientific, Madrid, Spain), freeze-dried, and stored until use. The purity and molecular weight of the polymer were routinely determined by sodium dodecyl sulphate polyacrylamide gel electrophoresis (SDS-PAGE) and mass spectrometry (MALDI-TOF/MS), obtaining a recombinamer of 120,353 Da. When necessary, (EIS)_2_-RGD6 was biotinylated using the EDC carbodiimide (BIE1308, Apollo Scientific, Stockport, UK) reaction with the addition of NHS (BIB101, Apollo Scientific, Stockport, UK), in order to increase the efficiency of the cross-linking procedure with Biotin-PEG6-Amine (BIPG1211, Apollo Scientific, Stockport, UK), following the manufacturer’s instructions. To prepare lyophilized (EIS)_2_-RGD6 for its administration, a solution of 50 mg/mL (EIS)_2_-RGD6 was performed in ice-cold PBS (70011044, Thermo Scientific, Paisley, UK) and subsequently incubated overnight at 4 °C, prior to its injection in the lesioned spinal cord.

### 2.2. Animals and Surgical Procedures

To perform the present study, a total of 92 adult female Wistar rats were used (three months of age; ~250 g). Animal housing and experimental procedures were carried out in accordance with the Spanish (Royal Decree 53/2013) and European Union (2010/63/EU) laws, and they were approved by the Bioethics Committee at The National Hospital of Paraplegics (Toledo, Spain) (Permit numbers 26/2019 and 27/2019). Spinal cord contusions were performed as previously reported by our group [53,54,55,56,57,58,59], with slight modifications. Briefly, a laminectomy was performed at the T10 spinal level in animals anesthetized by intraperitoneal injection of pentobarbital (Dolethal, 40 mg/kg; 07400060, Vetoquinol, Madrid, Spain) and xylacine (Xilagesic, 10 mg/kg; 26200021, Calier, Leon, Spain). Subsequently, the exposed spinal cord was subjected to a controlled impact of 200 kdynes using an Infinite Horizon Spinal Cord Impactor (IH-0400, Precision Systems and Instrumentation LLC, Fairfax, VA, USA). Intraparenchymal stereotaxic injection of vehicle (PBS) or (EIS)_2_-RGD6 (50 mg/mL) was performed immediately after spinal cord contusion. Please note that the specific injection points and volumes can be found in the Materials and Methods section named “Experimental design”. In all cases, stereotaxic injections were performed, at a rate of 1 μL/min, using a 33G needle and a Hamilton syringe attached to a microinjector (KDS-311, KD Scientific, Holliston, MA, USA) and a stereotaxic device (Kopf, Tujunga, CA, USA). At each injection point, the needle was maintained for a further four minutes, in order to minimize reflux of the solution. Except during the time of injection, the Hamilton syringe and the needle were maintained in ice, in order to avoid (EIS)_2_-RGD6 polymerization prior to its administration. As we have previously detailed [53,54,55,56,57,58,59], the post-operative care included subcutaneous injection of buprenorphine (0.03 mg/kg; 670588, Schering-Plough, Kenilworth, NJ, USA) on the day when the surgical process was conducted and at 1 day post-injury (dpi), and enrofloxacin (2.5 mg/kg; 27416, Bayer, Leverkusen, Germany) and saline solution (from 5 mL at 1 dpi to 1 mL at 5 dpi) during the first 5 dpi. The bladders were emptied daily until sacrifice or bladder function recovery. To ensure animal welfare, during the whole experimental process, the animals were inspected twice daily for signs of infection, autophagia, dehydration, or any other symptom of animal suffering by both the research staff involved in the present study as well as expert personnel from the animal facilities at the National Hospital for Paraplegia. Those animals that displayed any of the previously detailed symptoms were immediately sacrificed, following the recommendations given by the head veterinarian responsible of the Animal Facility and Experimental Surgery Unit at the National Hospital for Paraplegia.

### 2.3. Experimental Design

As a first essential step, we initially utilized an experimental set to determine the volumes and/or injection points for the intraparenchymal administration of (EIS)_2_-RGD6 [experiment (EIS)_2_-RGD6 I]. For this purpose, a total of 44 rats were divided into the following experimental groups: (1) Non-injected (NI) group, composed of NI-lesioned rats (*n* = 4); (2) Group PBS 2 + 2 + 2 µL, composed of lesioned rats injected with PBS at three injection points [1 mm rostral (2 µL), 0 mm (2 µL) and 1 mm caudal (2 µL) from epicentre, located in the medium line (0 mm lateral), and at 1 mm depth] (*n* = 6); (3) Group (EIS)_2_-RGD6 2 + 2 + 2 µL, composed of lesioned rats injected with (EIS)_2_-RGD6 using the same injection points and volumes detailed for group PBS 2 + 2 + 2 µL (*n* = 4); (4) Group PBS 6 µL, composed of lesioned rats injected with PBS at one injection point [0 mm from epicentre, 0 mm lateral, and 1 mm depth (6 µL)] (*n* = 5); (5) Group (EIS)_2_-RGD6 6 µL, composed of lesioned rats injected with (EIS)_2_-RGD6 using the same injection point and volume detailed for group PBS 6 µL (*n* = 5); (6) Group PBS 3 + 6 + 3 µL, composed of lesioned rats injected with PBS at three injection points [1 mm rostral (3 µL), 0 mm (6 µL) and 1 mm caudal (3 µL) from epicentre, located in the medium line (0 mm lateral) and at 1 mm depth] (*n* = 5); (7) Group (EIS)_2_-RGD6 3 + 6 + 3 µL, composed of lesioned rats injected with (EIS)_2_-RGD6 using the same injection points and volumes detailed for group PBS 3 + 6 + 3 µL (*n* = 6); (8) Group PBS 12 µL, composed of lesioned rats injected with PBS at one injection point [0 mm from epicentre, 0 mm lateral, and 1 mm depth (12 µL)] (*n* = 4); and (9) Group (EIS)_2_-RGD6 12 µL, composed of lesioned rats injected with (EIS)_2_-RGD6 using the same injection point and volume detailed for group PBS 12 µL (*n* = 5). In all cases, animals were sacrificed at 42 dpi. Based on the results obtained from the evaluation of motor functional recovery, myelin preservation, and mortality rate, an injection of 6 µL in the lesion epicentre was selected for the subsequent experimental sets.

We next conducted a second experimental set [experiment (EIS)_2_-RGD6 II], in order to evaluate the in vivo (EIS)_2_-RGD6 structure, distribution, degradation, porosity, and cell infiltration capacity. For this purpose, a total of 18 rats were subjected to spinal cord contusion and injected with 6 µL of biotinylated (EIS)_2_-RGD6 at one injection point (0 mm from epicentre, 0 mm lateral, and 1 mm depth). Animals were sacrificed at 1, 3, 7, 14, 28, and 42 dpi (*n* = 3 per group) for histological evaluation.

Finally, a third experimental set [experiment (EIS)_2_-RGD6 III] was carried out for deeper evaluation of the effects exerted by (EIS)_2_-RGD6 injection in motor functional recovery and various associated histopathological processes after SCI (myelin preservation, fibrosis, and astroglial and microglia/macrophage reactivity). Specifically, a total of 30 rats were sub-divided into the following experimental groups: (1) Group PBS 6 µL, composed of lesioned rats injected with PBS, which were sacrificed at 7 (*n* = 4) and 42 dpi (*n* = 9); and (2) Group (EIS)_2_-RGD6 6 µL, composed of lesioned rats injected with (EIS)_2_-RGD6, which were sacrificed at 7 (*n* = 6) and 42 dpi (*n* = 11). As previously stated, 6 µL of PBS or (EIS)_2_-RGD6 were injected into one injection point at 0 mm from epicentre, 0 mm lateral, and 1 mm depth in all animals in this experimental set.

### 2.4. Functional Analysis

#### 2.4.1. 21-Point Basso, Beattie, and Bresnahan Open-Field Test (BBB)

To evaluate motor functional recovery in experiments (EIS)_2_-RGD6 I and III, the BBB was carried out at 1, 5, 7, 14, 21, 28, 35, and 42 dpi, as previously described [53,54,55,56,57,59].

#### 2.4.2. Catwalk

Motor functional recovery in experiments (EIS)_2_-RGD6 I and (EIS)_2_-RGD6 III was further assessed using the Catwalk^®^ gait analysis system (version 7.1, Noldus, Wageningen, The Netherlands), as has been detailed in previous works performed by our group [57,59]. To this end, animals were assessed either before (to obtain pre-injury values; PI) or at the end of the study (42 dpi), in order to evaluate the following gait parameters: crossing velocity, regularity index, AB step patterns, front paws base of support (BOS), hind paws BOS, print positions, front paws stride length (SL), hind paws SL, and duty cycle (see [60] for a full description of the gait parameters analysed).

### 2.5. Histology

#### 2.5.1. Tissue Processing

As detailed in previous reports [53,54,55,56,57,58,59], animals were anesthetized as described above and sacrificed by intra-aortical perfusion of 1 mg/kg of 4% paraformaldehyde (PFA) (P6148, Sigma-Aldrich, Steinheim, Germany). Subsequently, a 2 cm spinal cord stretch containing the lesion was extracted, post-fixed in 4% PFA for 4 h (h), cryoprotected by immersion in 30% sucrose (84100, Sigma-Aldrich, Steinheim, Germany) for 72 h, frozen embedded in Neg-50 medium (6502, Epredia, Breda, The Netherlands), and cut using a cryostat to obtain parallel transverse spinal cord sections with a thickness of 30 µm, which were mounted on slides (J1800AMNZ, Thermo Scientific, Braunscweig, Germany) and stored at −20 °C until further use.

#### 2.5.2. Eriochrome Cyanine (Ecy) Staining

To quantify the amount of spared myelinated areas in animals used to perform experiments (EIS)_2_-RGD6 I and III, one set of parallel spinal cord sections per animal was processed for the visualization of spinal cord myelin by Ecy staining. The experimental protocol used has been previously described [54,57,59].

#### 2.5.3. Chromogen-Based Immunohistochemistry

To assess astroglial and microglia/macrophage reactivity in animals used to perform experiment (EIS)_2_-RGD6 III, one set of parallel spinal cord sections per animal was processed for immunohistochemical visualization of glial fibrillary acidic protein (GFAP) and ionized calcium-binding adaptor molecule 1 (Iba1), respectively. The chromogen-based simple immunohistochemistry protocol used for this purpose has been detailed in previous works [53,54,55,56,57,59]. The following primary and secondary antibodies were used: polyclonal goat anti-Iba1 (019-19741, Wako, Tokyo, Japan; 1:1000), monoclonal mouse anti-GFAP (G3893, Sigma-Aldrich, St. Louis, MO, USA; 1:1000), biotinylated goat anti-rabbit (BA1000, Vector, Newark, NJ; 1:500), biotinylated horse anti-mouse (BA2001, Vector, Newark, NJ, USA; 1:500), and horseradish peroxidase (HRP)-linked streptavidin (NEL750001EA, Perkin Elmer, Boston, MA, USA; 1:500). In all cases, and to confirm a lack of undesired cross-reactivity, sections processed without primary antibody were used as controls. No non-specific staining was observed.

#### 2.5.4. Fluorescence-Based Immunohistochemistry

To evaluate fibrosis in animals used to perform experiment (EIS)_2_-RGD6 III, one set of parallel spinal cord sections per animal was processed for the visualization of fibronectin (FN). To this end, we used the same fluorescence-based simple immunohistochemistry protocol that we have described in previous reports [57,59,61]. The following primary and secondary antibodies were used: polyclonal rabbit anti-FN (F3648, Sigma-Aldrich, Steinheim, Germany; 1:500) and Dylight594-linked goat anti-rabbit (ab96897, Abcam, Cambridge, UK). Again, sections processed without the primary antibody were used as controls, in order to confirm a lack of undesired cross-reactivity.

#### 2.5.5. (EIS)_2_-RGD6 Visualization

To visualize biotinylated (EIS)_2_-RGD6 in animals used to perform experiment (EIS)_2_-RGD6 II, a set of parallel sections per animal were first processed for the visualization of GFAP following the aforementioned fluorescence-based simple immunohistochemistry protocol. The following primary and secondary antibodies were used: monoclonal mouse anti-GFAP (G3893, Sigma-Aldrich, Steinheim, Germany; 1:500) and Dylight488-linked goat anti-mouse (ab96879, Abcam, Cambridge, UK). Subsequently, sections were immersed in buffer blocking—10% foetal bovine serum (10500064, Fisher Scientific, Madrid, Spain), 0.3% bovine serum albumin (A7906, Sigma Aldrich, Steinheim, Germany), 0.3% Triton X-100 (X100, Sigma Aldrich, Steinheim, Germany) in TBS—for 1 h at room temperature (RT), incubated with alexa594-linked streptavidin (S11227, Thermo Scientific, Paisley, UK; 1:500) for 1 h at RT and, after several washes, with DAPI (62247, Thermo Scientific, Paisley, UK; 1:5000) for 5 min at RT. Finally, sections were cover-slipped with Immumount (9990402, Epredia, Breda, The Netherlands).

### 2.6. Densitometric Analysis

To determine the amount of spared myelinated areas, the presence of astroglial and microglia/macrophages, and the degree of fibrosis, densitometric analysis was performed, as previously described [54,57,59], in those sets of parallel spinal cord sections processed by Ecy staining or GFAP, Iba1, and FN simple immunohistochemistries. Composite 10× images from whole Ecy stained sections corresponding to different rostro-caudal levels were obtained using a BX61 Motorized Research Microscope (Olympus, Barcelona, Spain) attached to a DP71 camera (Olympus, Barcelona, Spain), while composite 10× images obtained from whole sections processed for the visualization of GFAP, Iba1, and FN were obtained using an IX83 Motorized Microscope (Olympus, Barcelona, Spain) attached to an Orca 4.0 camera (Hamamatsu, Hamamatsu, Japan). All densitometric analyses were performed using the Fiji software (version 1.53q). Succinctly, spinal cord sections were carefully delineated and the total spinal cord section area was quantified. Subsequently, a threshold was set, according to the histological signal, and the Ecy, GFAP, Iba1, or FN-positive areas were quantified. Please note that the selected thresholds were maintained throughout the whole analysis of all images processed by the same histological procedure.

Similarly, to quantify (EIS)_2_-RGD6 degradation in experiment (EIS)_2_-RGD6 II, composite 10× images of whole sections processed for the visualization of biotinylated (EIS)_2_-RGD6 were obtained using an IX83 Motorized Microscope (Olympus, Barcelona, Spain) attached to an Orca 4.0 camera (Hamamatsu, Hamamatsu, Japan). Again, a threshold was selected according to the histological signal, which was maintained throughout the whole analysis, in order to determine the area occupied by (EIS)_2_-RGD6 at each analysed rostro-caudal level. (EIS)_2_-RGD6 volume estimation was carried out as previously described [62]. Briefly, the area occupied by (EIS)_2_-RGD6 in each serial section was measured as described above. The volume occupied by (EIS)_2_-RGD6 between successive serial sections was estimated from the area measurements and the known distance between sections (600 µm) and summed to estimate the total spinal cord volume occupied by (EIS)_2_-RGD6.

### 2.7. Porosity Analysis

To quantify (EIS)_2_-RGD6 porosity, composite 20× images of the whole porous (EIS)_2_-RGD6 scaffolds were obtained, from sections processed for the visualization of biotinylated (EIS)_2_-RGD6 and corresponding to the lesion epicentre (point of injection), using a TCS SP5 Resonant Scanner confocal microscope (Leica Microsystems, Mannheim, Germany). The confocal plane showing the higher presence of (EIS)_2_-RGD6 was selected to perform the analysis using the Fiji software (version 1.53q). Briefly, the porous scaffold of (EIS)_2_-RGD6 was carefully delineated and the area that it occupied was quantified. Subsequently, a threshold was selected according to the histological signal, in order to specifically identify the area occupied by pores. The selected threshold was maintained throughout the whole analysis. Finally, the analyse particles tool of the Fiji software was used to determine the number of pores in the porous scaffold of (EIS)_2_-RGD6, as well as the area of each detected pore.

### 2.8. Cell Count

The evaluation of cell infiltration in (EIS)_2_-RGD6 was performed in the same images and confocal planes used to evaluate (EIS)_2_-RGD6 porosity. Succinctly, the porous scaffold of (EIS)_2_-RGD6 was carefully delineated and the obtained selection was applied in the equivalent confocal plane showing DAPI nuclear counterstaining. Finally, the number of cell nuclei that were present in the porous scaffold of (EIS)_2_-RGD6 was quantified using the analyse particle tools of the Fiji software (version 1.53q).

### 2.9. Statistical Analysis

Two-way ANOVA followed by the Bonferroni post-hoc test was used to determine the potential existence of statistically significant between-group differences in (1) BBB score, sub-score, and specific sub-score parameters; (2) spared white matter; (3) (EIS)_2_-RGD6 pore size distribution; (4) the presence of astroglial cells; (5) the presence of microglia/macrophages; and (6) the degree of fibrosis. One-way ANOVA followed by the Bonferroni post-hoc test was conducted to determine the potential existence of statistically significant between-group differences in (1) Catwalk^®^ gait parameters (except for those cases comparing only two experimental groups, for which a two-tailed Student’s *t*-test was used); (2) pore density; (3) mean pore area; (4) median pore area; and (5) (EIS)_2_-RGD6 cell infiltration. Finally, Fisher’s exact test was used to determine the potential existence of statistically significant between-group differences in mortality in experiment (EIS)_2_-RGD6 I. These statistical tests were performed using GraphPad Prism 6 software and, in all cases, *p* < 0.05 was considered statistically significant.

## 3. Results

### 3.1. Determination of (EIS)_2_-RGD6 Injection Points and Volumes after SCI

As previously stated, we carried out a first experimental set [experiment (EIS)_2_-RGD6 I] to optimize the number of injections and volumes for the intraparenchymal administration of (EIS)_2_-RGD6 in the lesioned rat spinal cord. For this purpose, different injection systems were used (6 µL, 2 + 2 + 2 µL, 3 + 6 + 3 µL, and 12 µL; for details, see Section 2 subsection named “Experimental design”), and motor functional recovery, white matter preservation, and mortality rate were evaluated.

#### 3.1.1. Assessment of Motor Functional Recovery

**Figure 1:**
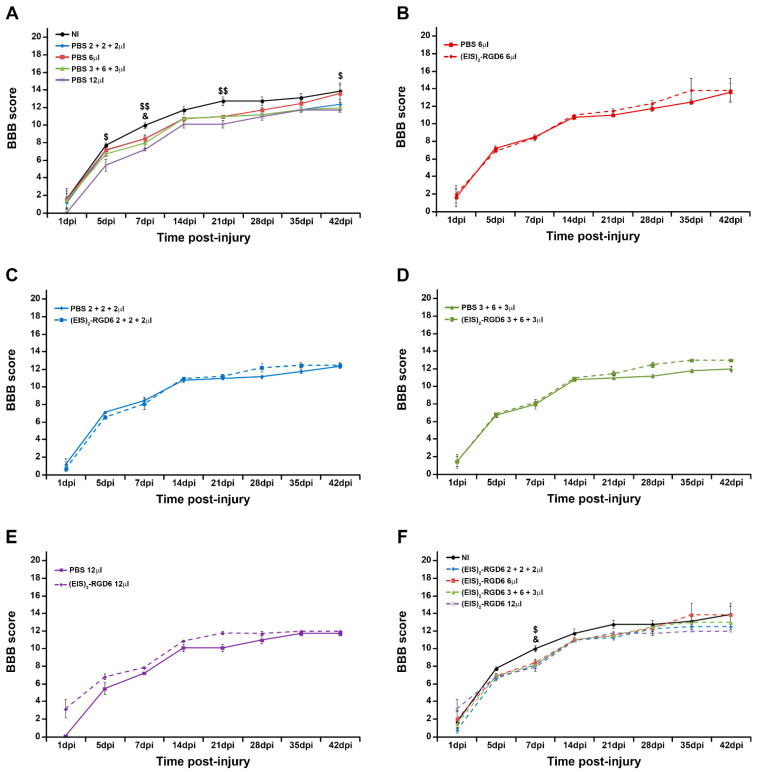
Determination of the injection system: 21-point Basso, Beattie, and Bresnahan open-field test (BBB) score. Graphs show data obtained from the motor functional recovery analysis using the BBB score in the experimental set performed to determine the best (EIS)_2_-RGD6 injection system [experiment (EIS)_2_-RGD6 I]. The BBB score was assessed at 1, 5, 7, 14, 21, 28, 35, and 42 days post-injury (dpi). The following comparisons are shown: (**A**) Non-injected (NI) group vs. PBS-injected groups (PBS 6 µL, PBS 2 + 2 + 2 µL, PBS 3 + 6 + 3 µL, or PBS 12 µL) (&, *p* < 0.05 NI vs. PBS 3 + 6 + 3 µL; $, *p* < 0.05 and $$, *p* < 0.01 NI vs. PBS 12 µL); (**B**) PBS 6 µL group vs. (EIS)_2_-RGD6 6 µL group; (**C**) PBS 2 + 2 + 2 µL group vs. (EIS)_2_-RGD6 2 + 2 + 2 µL group; (**D**) PBS 3 + 6 + 3 µL group vs. (EIS)_2_-RGD6 3 + 6 + 3 µL group; (**E**) PBS 12 µL group vs. (EIS)_2_-RGD6 12 µL group; and (**F**) NI group vs. (EIS)_2_-RGD6-injected groups [(EIS)_2_-RGD6 6 µL, (EIS)_2_-RGD6 2 + 2 + 2 µL, (EIS)_2_-RGD6 3 + 6 + 3 µL or (EIS)_2_-RGD6 12 µL] [&, *p* < 0.05 NI vs. (EIS)_2_-RGD6 3 + 6 + 3 µL; $, *p* < 0.05 NI vs. (EIS)_2_-RGD6 12 µL]. In (**A**–**F**), the potential existence of statistically significant between-group differences was assessed by two-way ANOVA followed by the Bonferroni post-hoc test. Please note that data obtained from the separate analysis of the individual BBB parameters evaluated can be found in Table S1.

BBB score evaluation initially showed that intraparenchymal PBS administration in all tested injection systems induced a slight non-significant decrease in motor function recovery, when compared to the NI group, reaching statistical significance at specific time points in groups PBS 3 + 6 + 3 µL (7 dpi) and PBS 12 µL (5, 7, and 21 dpi); see Figure 1A. Notably, from all injection systems and volumes tested, those animals that received a single injection of 6 µL of PBS in the lesion epicentre displayed the most similar motor functional recovery to that of NI animals, being almost indistinguishable at the end of the study (42 dpi); see Figure 1A. Moreover, we found that, independent of the injection system and volume used, (EIS)_2_-RGD6 administration did not induce significant changes in BBB score, in comparison with the corresponding PBS-injected control groups (Figure 1B–E). Finally, similarly to what was observed in PBS-injected groups, when comparing the different groups injected with (EIS)_2_-RGD6 with the NI control group, all analysed injection systems led to a slight non-significant trend of diminished BBB score, reaching statistical significance in groups (EIS)_2_-RGD6 3 + 6 + 3 µL and (EIS)_2_-RGD6 12 µL at 7 dpi (Figure 1F). As can be seen from the figure, the group (EIS)_2_-RGD6 6 µL again exhibited the most similar BBB score progression to that observed in the NI control group, displaying an almost identical BBB score at 42 dpi (Figure 1F).

**Figure 2:**
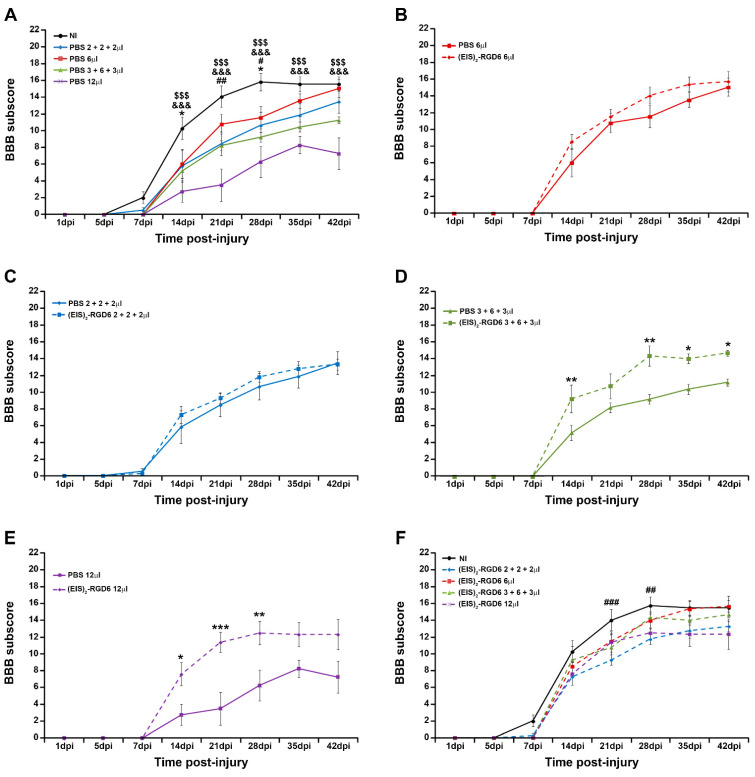
Determination of the injection system: 21-point Basso, Beattie, and Bresnahan open-field test (BBB) sub-score. Graphs show data obtained from the analysis of motor functional recovery using the BBB sub-score in the experimental set performed to determine the best (EIS)_2_-RGD6 injection system [experiment (EIS)_2_-RGD6 I]. The BBB sub-score was assessed at 1, 5, 7, 14, 21, 28, 35, and 42 days post-injury (dpi). The following comparisons are shown: (**A**) Non-injected (NI) group vs. PBS-injected groups (PBS 6 µL, PBS 2 + 2 + 2 µL, PBS 3 + 6 + 3 µL, or PBS 12 µL) (*, *p* < 0.05 NI vs. PBS 6 µL; #, *p* 0.05 and ##, *p* < 0.01 NI vs. PBS 2 + 2 + 2 µL; &&&, *p* < 0.001 NI vs. PBS 3 + 6 + 3 µL; $$$, *p* < 0.001 vs. PBS 12 µL); (**B**) PBS 6 µL group vs. (EIS)_2_-RGD6 6 µL group; (**C**) PBS 2 + 2 + 2 µL group vs. (EIS)_2_-RGD6 2 + 2 + 2 µL group; (**D**) PBS 3 + 6 + 3 µL group vs. (EIS)_2_-RGD6 3 + 6 + 3 µL group [*, *p* < 0.05 and **, *p* < 0.01 PBS 3 + 6 + 3 µL vs. (EIS)_2_-RGD6 3 + 6 + 3 µL]; (**E**) PBS 12 µL group vs. (EIS)_2_-RGD6 12 µL group (*, *p* < 0.05; **, *p* < 0.01 and ***, *p* < 0.001 PBS 12 µL vs. (EIS)_2_-RGD6 12 µL); and (**F**) NI group vs. (EIS)_2_-RGD6-injected groups [(EIS)_2_-RGD6 6 µL, (EIS)_2_-RGD6 2 + 2 + 2 µL, (EIS)_2_-RGD6 3 + 6 + 3 µL, or (EIS)_2_-RGD6 12 µL] [##, *p* < 0.01 and ###, *p* < 0.001 NI vs. (EIS)_2_-RGD6 2 + 2 + 2 µL]. In (**A**–**F**), the potential existence of statistically significant between-group differences was assessed by two-way ANOVA followed by the Bonferroni post-hoc test. Please note that data obtained from the separate analysis of the individual BBB parameters evaluated can be found in Table S1.

In accordance with the previously detailed observations, evaluation of the BBB sub-score showed that, independent of the injection system, intraparenchymal PBS injection induced a significant decrease in motor functional recovery, when compared to the NI control group (NI vs. PBS 6 µL: 14 and 28 dpi; NI vs. PBS 2 + 2 + 2 µL: 21 and 28 dpi; NI vs. PBS 3 + 6 + 3 µL: 14, 21, 28, 35 and 42 dpi; NI vs. PBS 12 µL: 14, 21, 28, 35 and 42 dpi); see Figure 2A. Interestingly, an evident relationship was observed between increasing injection points and/or volumes and a more prominent impairment in functional recovery, with the PBS 6 µL group again being the one that showed a more similar progression in BBB sub-score than that observed in the NI control group (Figure 2A). Once more, we did not find significant differences in groups (EIS)_2_-RGD6 6 µL and (EIS)_2_-RGD6 2 + 2 + 2 µL, when compared with their corresponding PBS-injected control groups (Figure 2B,C). However, in contrast to what was observed in the BBB score, groups (EIS)_2_-RGD6 3 + 6 + 3 µL and (EIS)_2_-RGD6 12 µL displayed significantly higher BBB sub-scores at different evaluated time points than those observed in their corresponding control groups [14, 28, 35, and 42 dpi for group (EIS)_2_-RGD6 3 + 6 + 3 µL, Figure 2D; 14, 21 and 28 dpi for group (EIS)_2_-RGD6 12 µL, Figure 2E], an apparent discrepancy that can be explained by the intrinsic limitations of the BBB score. Nevertheless, when the different groups injected with (EIS)_2_-RGD6 were compared to the NI control group, all analysed injection systems led to a slight non-significant impairment in motor functional recovery, which reached statistical significance in the (EIS)_2_-RGD6 2 + 2 + 2 µL group at 21 and 28 dpi (Figure 2F). Again, animals that received a single injection of 6 µL of (EIS)_2_-RGD6 in the lesion epicentre showed the most similar BBB sub-score progression to that observed in NI animals (Figure 2F; data obtained from the separate analysis of the individual BBB parameters evaluated can be found in Table S1).

**Figure 3:**
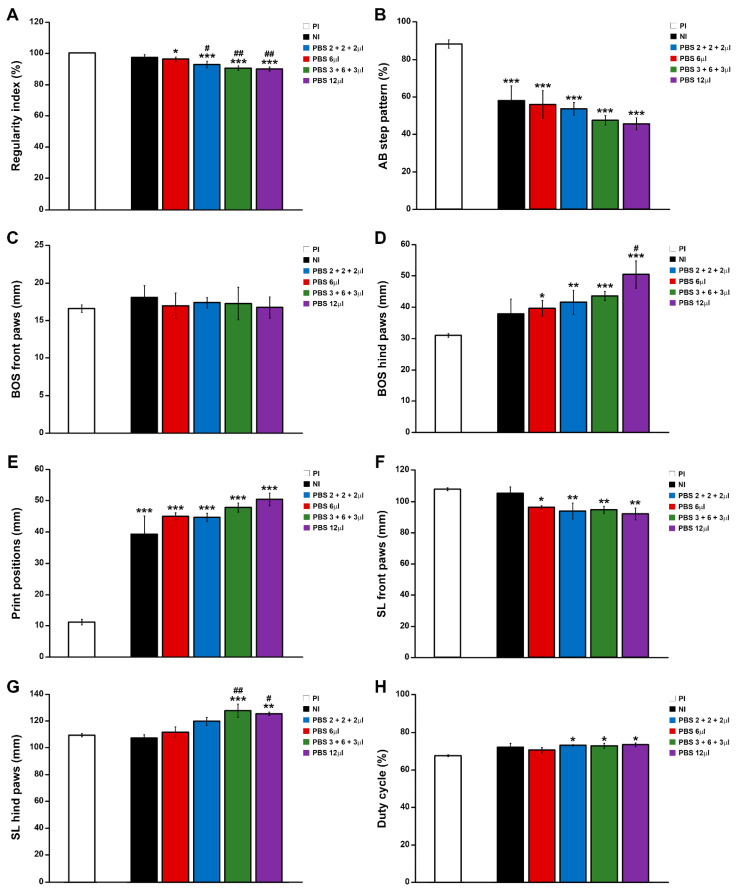
Determination of the injection system: Catwalk^®^-based gait analysis I. Graphs present data obtained from the evaluation at 42 days post-injury of motor functional recovery using the Catwalk^®^ gait analysis system [non-injected (NI) group vs. PBS-injected groups (PBS 6 µL, PBS 2 + 2 + 2 µL, PBS 3 + 6 + 3 µL, or PBS 12 µL)] in the experimental set performed to determine the best (EIS)_2_-RGD6 injection system [experiment (EIS)_2_-RGD6 I]. The following gait parameters were evaluated: (**A**) regularity index; (**B**) AB step pattern; (**C**) front paws base of support (BOS); (**D**) hind paws BOS; (**E**) print positions; (**F**) front paws stride length (SL); (**G**) hind paws SL; and (**H**) duty cycle. In (**A**–**H**), the potential existence of statistically significant between-group differences was assessed by one-way ANOVA followed by the Bonferroni post-hoc test. In all cases: *, *p* < 0.05; **, *p* < 0.01; and *** *p* < 0.001 vs. pre-injury (PI), while #, *p* < 0.05 and ##, *p* < 0.01 vs. NI.

**Figure 4:**
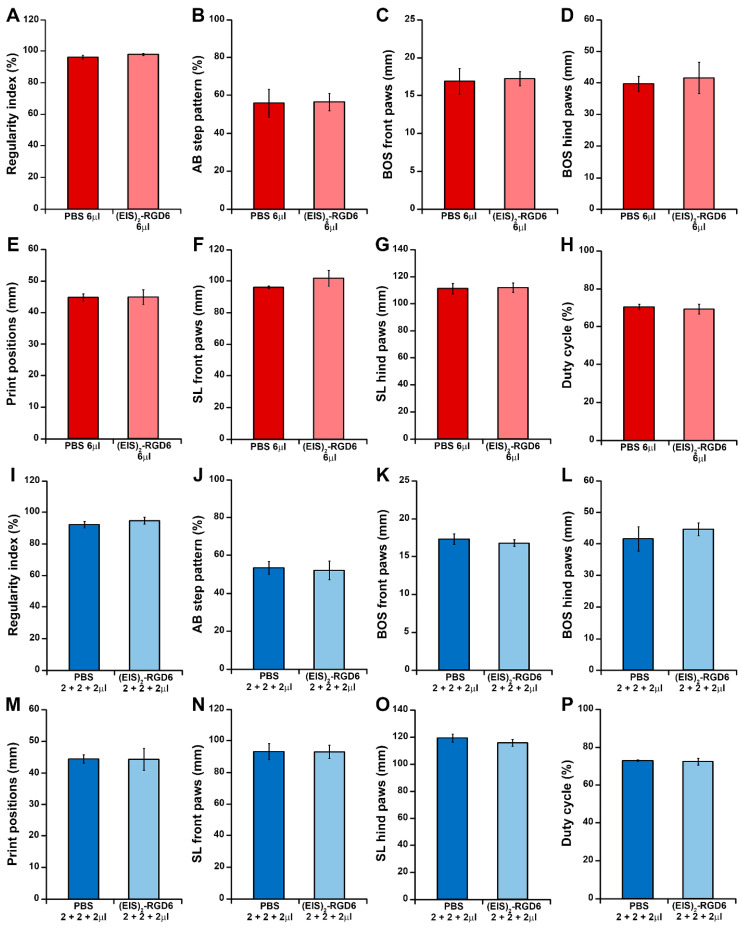
Determination of the injection system: Catwalk^®^-based gait analysis II. Graphs show data obtained from the evaluation at 42 days post-injury of motor functional recovery using the Catwalk^®^ gait analysis system [PBS 6 µL group vs. (EIS)_2_-RGD6 6 µL group (**A**–**H**) and PBS 2 + 2 + 2 µL group vs. (EIS)_2_-RGD6 2 + 2 + 2 µL group (**I**–**P**)] in the experimental set performed to determine the best (EIS)_2_-RGD6 injection system [experiment (EIS)_2_-RGD6 I]. The following gait parameters were evaluated: (**A**,**I**) regularity index; (**B**,**J**) AB step pattern; (**C**,**K**) front paws base of support (BOS); (**D**,**L**) hind paws BOS; (**E**,**M**) print positions; (**F**, **N**) front paws stride length (SL); (**G**,**O**) hind paws SL; and (**H**,**P**) duty cycle. In (**A**–**P**), the potential existence of statistically significant between-group differences was assessed by two-tailed Student’s *t*-test.

**Figure 5:**
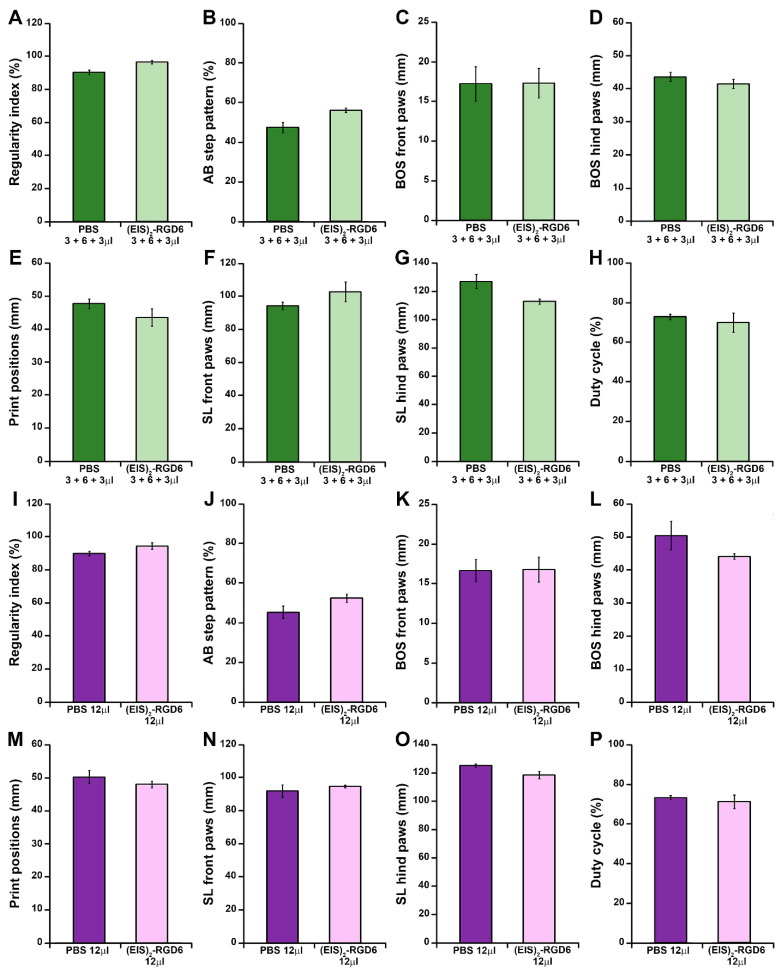
Determination of the injection system: Catwalk^®^-based gait analysis III. Figure showing data obtained from the evaluation at 42 days post-injury of motor functional recovery using the Catwalk^®^ gait analysis system [PBS 3 + 6 + 3 µL group vs. (EIS)_2_-RGD6 3 + 6 + 3 µL group (**A**–**H**) and PBS 12 µL group vs. (EIS)_2_-RGD6 12 µL group (**I**–**P**)] in the experimental set performed to determine the best (EIS)_2_-RGD6 injection system [experiment (EIS)_2_-RGD6 I]. The following gait parameters were evaluated: (**A**,**I**) regularity index; (**B**,**J**) AB step pattern; (**C**,**K**) front paws base of support (BOS); (**D**,**L**) hind paws BOS; (**E**,**M**) print positions; (**F**,**N**) front paws stride length (SL); (**G**,**O**) hind paws SL; and (**H**,**P**) duty cycle. In (**A**–**P**), the potential existence of statistically significant between-group differences was assessed by two-tailed Student’s *t*-test.

**Figure 6:**
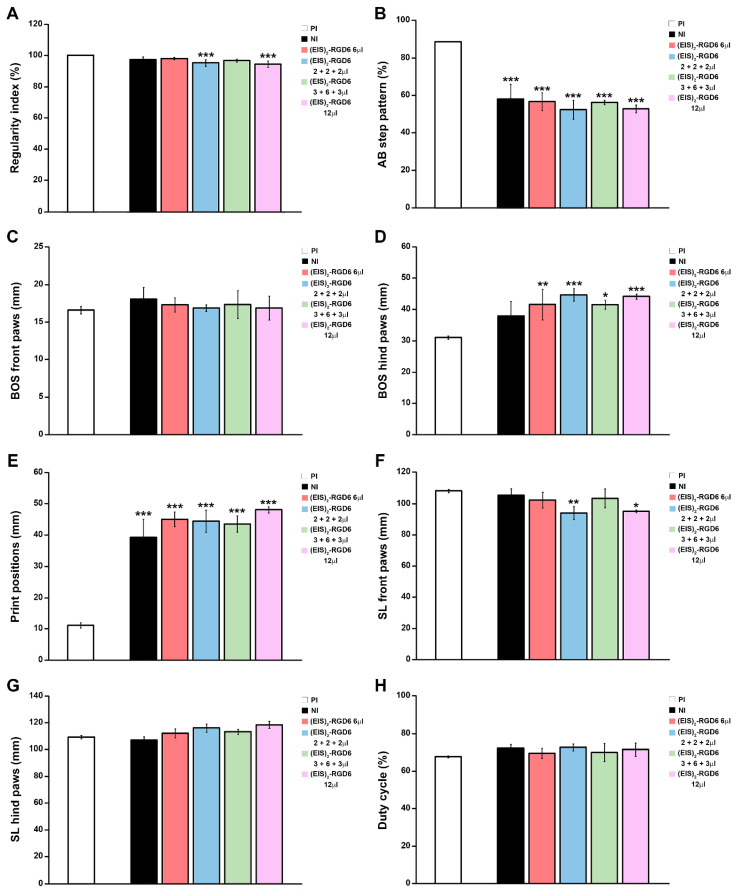
Determination of the injection system: Catwalk^®^-based gait analysis IV. Graphs show data obtained from the evaluation at 42 days post-injury of motor functional recovery using the Catwalk^®^ gait analysis system [NI group vs. (EIS)_2_-RGD6-injected groups [(EIS)_2_-RGD6 6 µL, (EIS)_2_-RGD6 2 + 2 + 2 µL, (EIS)_2_-RGD6 3 + 6 + 3 µL, or (EIS)_2_-RGD6 12 µL)] in the experimental set performed to determine the best (EIS)_2_-RGD6 injection system [experiment (EIS)_2_-RGD6 I]. The following gait parameters were evaluated: (**A**) regularity index; (**B**) AB step pattern; (**C**) front paws base of support (BOS); (**D**) hind paws BOS; (**E**) print position; (**F**) front paws stride length (SL); (**G**) hind paws SL; and (**H**), duty cycle. In (**A**–**H**), the potential existence of statistically significant between-group differences was assessed by one-way ANOVA followed by the Bonferroni post-hoc test. In all cases: *, *p* < 0.05; **, *p* < 0.01; and ***, *p* < 0.001 vs. pre-injury (PI).

Finally, motor functional recovery was also evaluated using the Catwalk^®^ gait analysis system at the end of the study (42 dpi). In agreement with the previously detailed observations, when compared to the NI control group, we observed an evident relationship between increasing PBS injection points and/or volumes and a non-significant trend of worsening in different gait parameters, such as regularity index (Figure 3A), AB step pattern (Figure 3B), hind paws BOS (Figure 3D), print positions (Figure 3E), front paws SL (Figure 3F), hind paws SL (Figure 3G), and duty cycle (Figure 3H), reaching statistical significance in regularity index (PBS 2 + 2 + 2 µL, PBS 3 + 6 + 3 µL, and PBS 12 µL groups; Figure 3A), hind paws BOS (PBS 12 µL group; Figure 3D), and hind paws SL (PBS 3 + 6 + 3 µL and PBS 12 µL groups; Figure 3G). Interestingly, the PBS 6 µL group was the only experimental group that did not show statistically significant differences in any of the evaluated gait parameters, when compared to the NI control group, again being the experimental group that displayed a more similar motor function performance to that observed in NI rats at 42 dpi (Figure 3). In addition, we also determined that, independent of the injection system used, (EIS)_2_-RGD6 administration did not significantly vary any of the gait parameters evaluated, when compared to the corresponding PBS-injected control groups (Figure 4 and Figure 5). Finally, comparison of the different (EIS)_2_-RGD6-injected groups with the NI control group did not show significant differences in any of the analysed gait parameters (Figure 6). However, it is interesting to note that, when compared to that observed in PI values, the (EIS)_2_-RGD6 2 + 2 + 2 µL and (EIS)_2_-RGD6 12 µL groups displayed a significantly lower regularity index (Figure 6A) and front paws SL (Figure 6F). However, equivalently to what was observed in the NI control group, both (EIS)_2_-RGD6 6 µL and (EIS)_2_-RGD6 3 + 6 + 3 µL groups did not, thereby supporting the conclusion that these two experimental groups exhibited motor functional performance that was more similar to that observed in NI rats.

#### 3.1.2. Evaluation of White Matter Preservation

**Figure 7:**
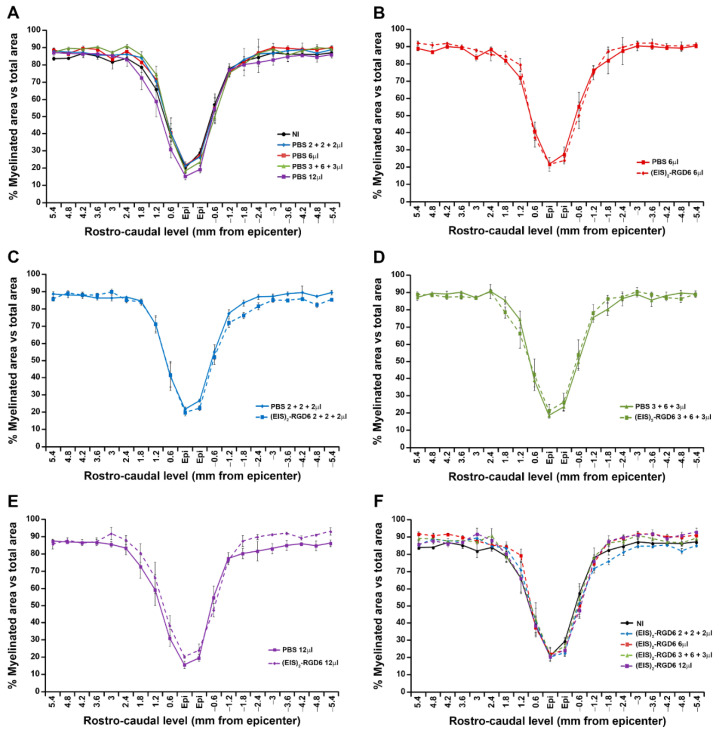
Determination of the injection system: Myelin preservation. Graphs show data obtained from the densitometrical analysis at 42 days post-injury of myelin preservation in eriochrome cyanine-stained sections from the experimental set performed to determine the best (EIS)_2_-RGD6 injection system [experiment (EIS)_2_-RGD6 I]. The following comparisons are shown: (**A**) Non-injected (NI) group vs. PBS-injected groups (PBS 6 µL, PBS 2 + 2 + 2 µL, PBS 3 + 6 + 3 µL, or PBS 12 µL); (**B**) PBS 6 µL group vs. (EIS)_2_-RGD6 6 µL group; (**C**) PBS 2 + 2 + 2 µL group vs. (EIS)_2_-RGD6 2 + 2 + 2 µL group; (**D**) PBS 3 + 6 + 3 µL group vs. (EIS)_2_-RGD6 3 + 6 + 3 µL group; (**E**) PBS 12 µL group vs. (EIS)_2_-RGD6 12 µL group; and (**F**) NI group vs. (EIS)_2_-RGD6-injected groups [(EIS)_2_-RGD6 6 µL, (EIS)_2_-RGD6 2 + 2 + 2 µL, (EIS)_2_-RGD6 3 + 6 + 3 µL, or (EIS)_2_-RGD6 12 µL]. In (**A**–**F**), the potential existence of statistically significant between-group differences was assessed by two-way ANOVA followed by the Bonferroni post-hoc test. Please note that representative images from Ecy stained sections corresponding to each evaluated group can be found in Figure S1.

To further determine the best injection system, we subsequently quantified the amount of spared white matter. As shown in Figure 7, neither the comparison of the different PBS-injected groups with the NI group (Figure 7A), the comparison of each (EIS)_2_-RGD6-injected group with its corresponding PBS-injected control group (Figure 7B–E), nor the comparison of the different (EIS)_2_-RGD6-injected groups with the NI control group (Figure 7F) showed any statistically significant change (representative images from Ecy stained sections corresponding to each evaluated group can be found in Figure S1).

#### 3.1.3. Mortality

**Figure 8:**
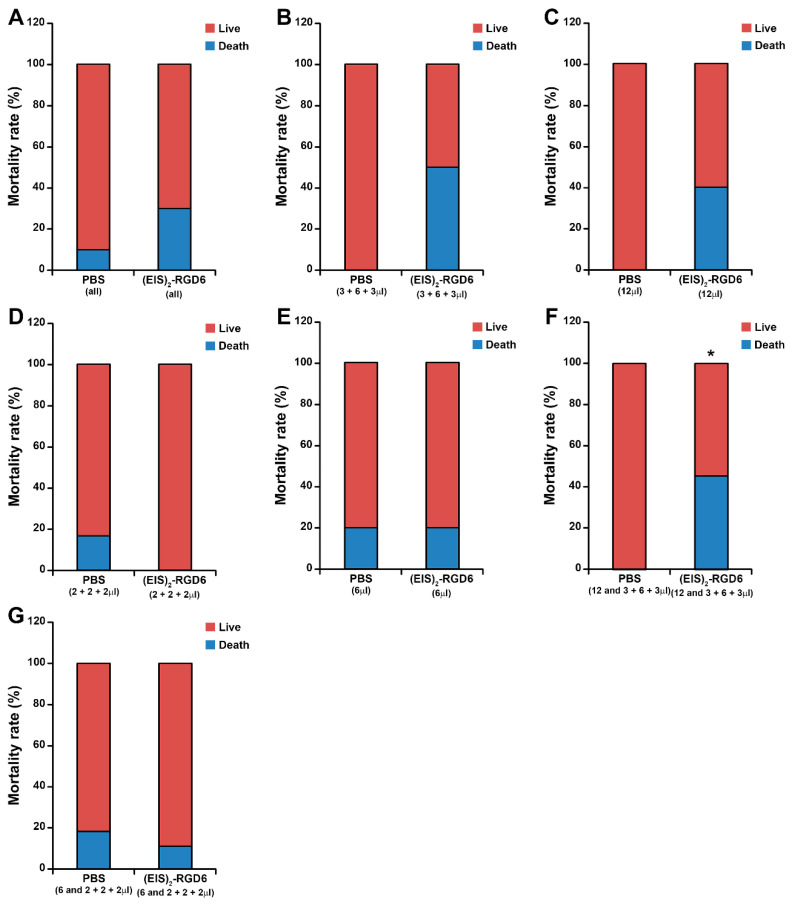
Determination of the injection system: Mortality rate. Graphs show data obtained from the evaluation of mortality rate in animals from the experimental set performed to determine the best (EIS)_2_-RGD6 injection system [experiment (EIS)_2_-RGD6 I]. The following comparisons were performed: (**A**) All PBS-injected animals (PBS 6 µL, PBS 2 + 2 + 2 µL, PBS 3 + 6 + 3 µL, and PBS 12 µL groups) vs. all (EIS)_2_-RGD6-injected animals [(EIS)_2_-RGD6 6 µL, (EIS)_2_-RGD6 2 + 2 + 2 µL, (EIS)_2_-RGD6 3 + 6 + 3 µL, and (EIS)_2_-RGD6 12 µL]; (**B**) PBS 3 + 6 + 3 µL group vs. (EIS)_2_-RGD6 3 + 6 + 3 µL group; (**C**) PBS 12 µL group vs. (EIS)_2_-RGD6 12 µL group; (**D**) PBS 2 + 2 + 2 µL group vs. (EIS)_2_-RGD6 2 + 2 + 2 µL group; (**E**) PBS 6 µL group vs. (EIS)_2_-RGD6 6 µL group; (**F**) all animals injected with 12 µL of PBS (PBS 3 + 6 + 3 µL and PBS 12 µL groups) vs. all animals injected with 12 µL of (EIS)_2_-RGD6 [(EIS)_2_-RGD6 3 + 6 + 3 µL and (EIS)_2_-RGD6 12 µL groups] (*, *p* < 0.05); and (**G**) all animals injected with 6 µL of PBS (PBS 2 + 2 + 2 µL and PBS 6 µL groups) vs. all animals injected with 6 µL of (EIS)_2_-RGD6 [(EIS)_2_-RGD6 2 + 2 + 2 µL and (EIS)_2_-RGD6 6 µL groups]. In (**A**–**G**), the potential existence of statistically significant between-group differences was assessed by Fisher’s exact test. Please note that contingency tables corresponding to the analysis of mortality can be found in Table S2.

Finally, regarding the analysis of mortality, we first found an evident non-significant trend of increased mortality rate associated with (EIS)_2_-RGD6 administration when all PBS-injected animals (independent of the injection system) were compared to all (EIS)_2_-RGD6-injected animals (independent of the injection system); see Figure 8A. Further evaluation of this intriguing observation showed that both (EIS)_2_-RGD6 3 + 6 + 3 µL (Figure 8B) and (EIS)_2_-RGD6 12 µL (Figure 8C) groups showed an evident non-significant trend of increased mortality rate, compared to that observed in their corresponding PBS-injected controls, while the (EIS)_2_-RGD6 2 + 2 + 2 µL (Figure 8D) and (EIS)_2_-RGD6 6 µL (Figure 8E) groups did not, thereby suggesting that (EIS)_2_-RGD6 volume—and not the number of injections performed—may influence this parameter. Accordingly, we found that those animals injected with 12 µL of (EIS)_2_-RGD6 [(EIS)_2_-RGD6 3 + 6 + 3 µL and (EIS)_2_-RGD6 12 µL groups] displayed a statistically significant increase in mortality, when compared with their PBS-injected counterparts (Figure 8F)—an effect that completely disappeared in animals injected with 6 µL of (EIS)_2_-RGD6 [(EIS)_2_-RGD6 2 + 2 + 2 µL and (EIS)_2_-RGD6 6 µL] (Figure 8G). Contingency tables corresponding to the analysis of mortality can be found in Table S2.

Based on the previously detailed observations, we decided to select a single injection of 6 µL of (EIS)_2_-RGD6 in the lesion epicentre to perform the subsequent experimental sets, as animals subjected to this injection system showed the closest motor functional recovery to that observed in the NI control group, did not show significant variations in the amount of spared white matter, and did not present an altered mortality rate.

### 3.2. Evaluation of (EIS)_2_-RGD6 Distribution and Structure after SCI

Subsequently, a second experimental set [experiment (EIS)_2_-RGD6 II] was conducted to determine the temporal evolution of the distribution, degradation, porosity, and cell infiltration of (EIS)_2_-RGD6. For this purpose, animals were injected—following the injection system previously selected—with biotinylated (EIS)_2_-RGD6 and sacrificed at 1, 3, 7, 14, 28, or 42 dpi for histological evaluation.

#### 3.2.1. Qualitative Microscopic Analysis

**Figure 9:**
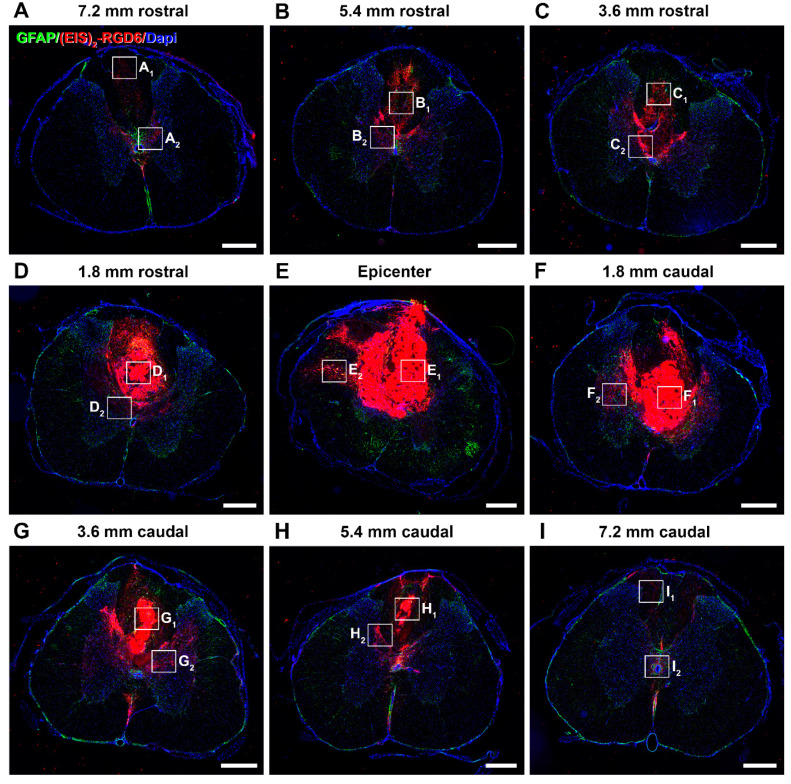
Qualitative microscopic analysis of (EIS)_2_-RGD6 distribution at 1 day post-injury. Representative images from whole spinal cord sections processed for the visualization of biotinylated (EIS)_2_-RGD6 and glial fibrillary acidic protein (GFAP) at 1 day post-injury [experiment (EIS)_2_-RGD6 II]. The following rostro-caudal spinal cord levels are shown: (**A**) 7.2 mm rostral; (**B**) 5.4 mm rostral; (**C**) 3.6 mm rostral; (**D**) 1.8 mm rostral; (**E**) 0 mm; (**F**) 1.8 mm caudal; (**G**) 3.6 mm caudal; (**H**) 5.4 mm caudal; and (**I**) 7.2 mm caudal from epicentre. Scale bars, 500 µm. Please note that higher magnification images corresponding to the areas highlighted with squares in (**A**–**I**) can be found in Figure 11.

**Figure 10:**
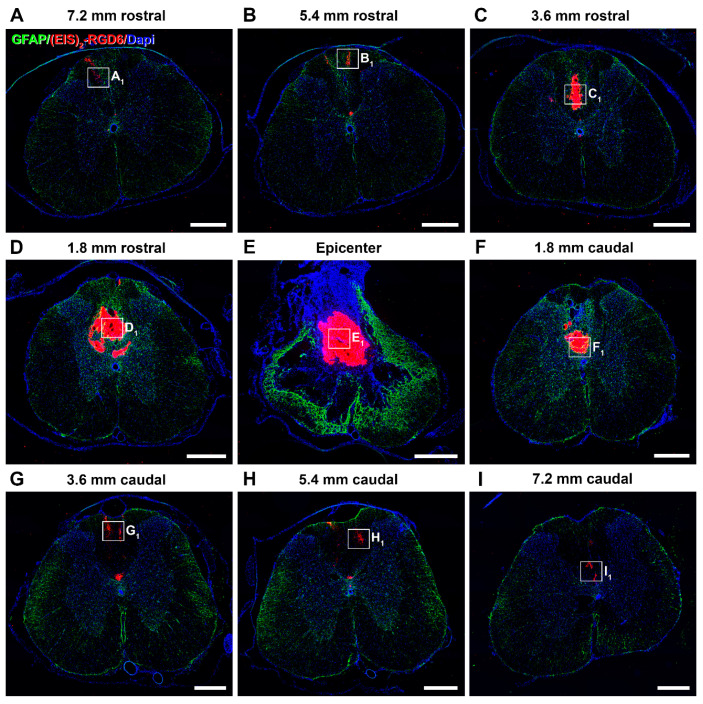
Qualitative microscopic analysis of (EIS)_2_-RGD6 distribution at 42 days post-injury. Representative images from whole spinal cord sections processed for the visualization of biotinylated (EIS)_2_-RGD6 and glial fibrillary acidic protein (GFAP) at 42 days post-injury [experiment (EIS)_2_-RGD6 II]. The following rostro-caudal spinal cord levels are shown: (**A**) 7.2 mm rostral; (**B**) 5.4 mm rostral; (**C**) 3.6 mm rostral; (**D**) 1.8 mm rostral; (**E**) 0 mm; (**F**) 1.8 mm caudal; (**G**) 3.6 mm caudal; (**H**) 5.4 mm caudal; and (**I**) 7.2 mm caudal from epicentre. Scale bars, 500 µm. Please note that higher magnification images corresponding to the areas highlighted with squares in (**A**–**I**) can be found in Figure 12.

**Figure 11:**
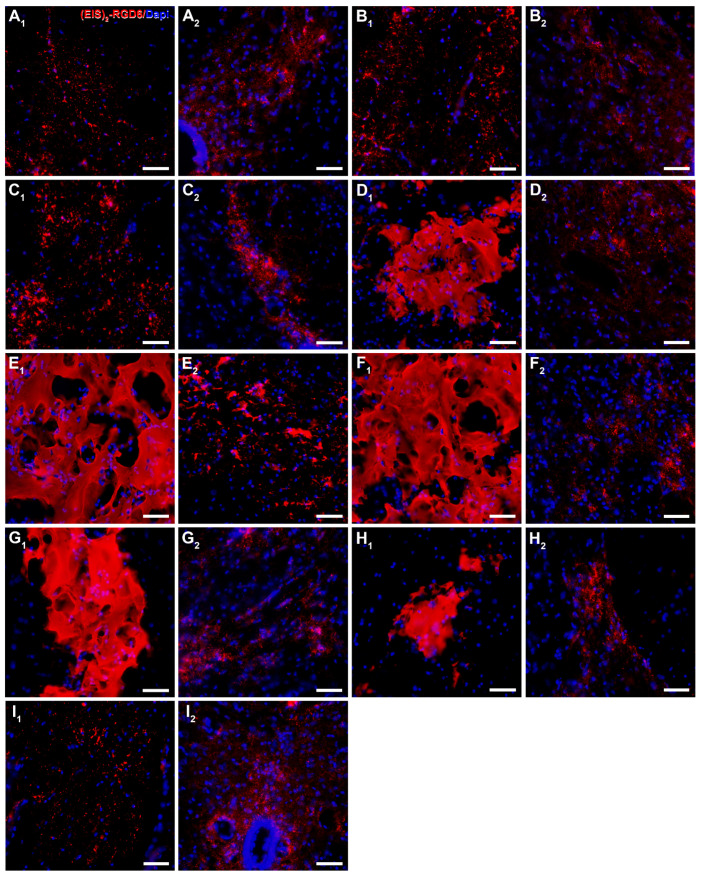
Qualitative microscopic analysis of (EIS)_2_-RGD6 structure at 1 day post-injury. Representative higher magnification images of spinal cord sections processed for the visualization of biotinylated (EIS)_2_-RGD6 at 1 day post-injury [experiment (EIS)_2_-RGD6 II], showing (EIS)_2_-RGD6 structure at the following representative rostro-caudal spinal cord levels: (**A_1_**,**A_2_**) 7.2 mm rostral; (**B_1_**,**B_2_**) 5.4 mm rostral; (**C_1_**,**C_2_**) 3.6 mm rostral; (**D_1_**,**D_2_**) 1.8 mm rostral; (**E_1_**,**E_2_**) 0 mm; (**F_1_**,**F_2_**) 1.8 mm caudal; (**G_1_**,**G_2_**) 3.6 mm caudal; (**H_1_**,**H_2_**) 5.4 mm caudal; and (**I_1_**,**I_2_**) 7.2 mm caudal from the injury epicentre. Scale bars, 50 µm. Please note that the specific areas shown in these images are highlighted in Figure 9.

**Figure 12:**
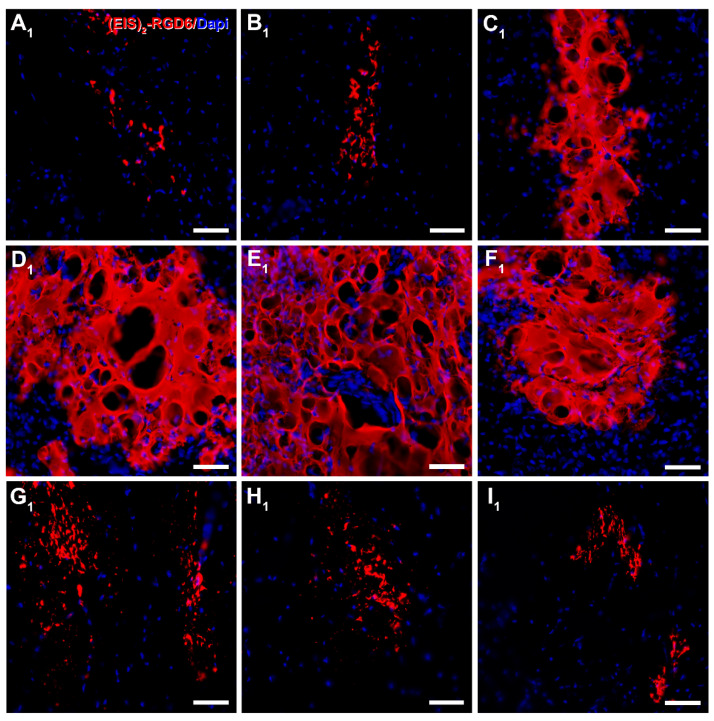
Qualitative microscopic analysis of (EIS)_2_-RGD6 structure at 42 days post-injury. Representative higher magnification images of spinal cord sections processed for the visualization of biotinylated (EIS)_2_-RGD6 at 42 days post-injury [experiment (EIS)_2_-RGD6 II], showing (EIS)_2_-RGD6 structure at the following representative rostro-caudal spinal cord levels: (**A_1_**) 7.2 mm rostral; (**B_1_**) 5.4 mm rostral; (**C_1_**) 3.6 mm rostral; (**D_1_**) 1.8 mm rostral; (**E_1_**) 0 mm; (**F_1_**) 1.8 mm caudal; (**G_1_**) 3.6 mm caudal; (**H_1_**) 5.4 mm caudal; and (**I_1_**) 7.2 mm caudal from the injury epicentre. Scale bars, 50 µm. Please note that the specific areas shown in these images are highlighted in Figure 10.

Regarding (EIS)_2_-RGD6 distribution, qualitative microscopic analysis of spinal cord sections processed for the visualization of (EIS)_2_-RGD6 demonstrated that, at all evaluated time points, (EIS)_2_-RGD6 was detected at all analysed rostro-caudal levels (from 7.2 mm rostral to 7.2 mm caudal to the lesion epicentre). Notably, the presence of (EIS)_2_-RGD6 showed an evident correlation with the lesioned areas at all analysed times post-SCI, as (EIS)_2_-RGD6 accumulation was higher in spinal cord sections corresponding to the lesion epicentre (point of injection) and at adjacent rostro-caudal levels, where (EIS)_2_-RGD6 was prominently observed in the centre of the lesion, compared to what was detected at rostro-caudal levels separated from the injury site, where (EIS)_2_-RGD6 was mainly found in the lesioned dorsal columns, in grey matter areas contacting them, and surrounding the central canal. Finally, an evident qualitative decrease in the presence of (EIS)_2_-RGD6 was observed from 1 to 7 dpi (mainly in the lesion epicentre and at the adjacent spinal cord levels), which was less obvious from 7 to 42 dpi. Representative images showing (EIS)_2_-RGD6 distribution in the lesioned spinal cord are shown in Figure 9 (1 dpi), Figure S2 (3 dpi), Figure S3 (7 dpi), Figure S4 (14 dpi), Figure S5 (28 dpi), and Figure 10 (42 dpi); while higher magnification images corresponding to the areas highlighted with squares in the previously detailed figures and supplementary files can be found in Figure 11 (1 dpi), Figure S6 (3 dpi), Figure S7 (7 dpi), Figure S8 (14 dpi), Figure S9 (28 dpi), and Figure 12 (42dpi), respectively.

On the other hand, at all analysed survival times, the (EIS)_2_-RGD6 structure displayed clear differences depending on the distance from the injection point. More specifically, in the lesion epicentre and at adjacent spinal cord levels (principally between 1.8 mm rostral and 1.8 mm caudal from the lesion epicentre), (EIS)_2_-RGD6 was mainly found as a porous scaffold, with infiltrated cells inside, surrounded by dispersed (EIS)_2_-RGD6 + fibres and/or particles. In contrast, at rostral and caudal spinal cord levels, the porous scaffold almost completely disappeared and (EIS)_2_-RGD6 was prominently observed as dispersed fibres and/or particles. Finally, qualitative microscopic analysis did not reveal evident differences in (EIS)_2_-RGD6 structure over the analysed time period. Representative images showing (EIS)_2_-RGD6 structure can be found in Figure 11 (1 dpi), Figure S6 (3 dpi), Figure S7 (7 dpi), Figure S8 (14 dpi), Figure S9 (28 dpi), and Figure 12 (42 dpi); while specific areas shown in these images at higher magnification are highlighted in Figure 9 (1 dpi), Figure S2 (3 dpi), Figure S3 (7 dpi), Figure S4 (14 dpi), Figure S5 (28 dpi), and Figure 10 (42 dpi), respectively.

#### 3.2.2. Quantification of (EIS)_2_-RGD6 Degradation

**Figure 13:**
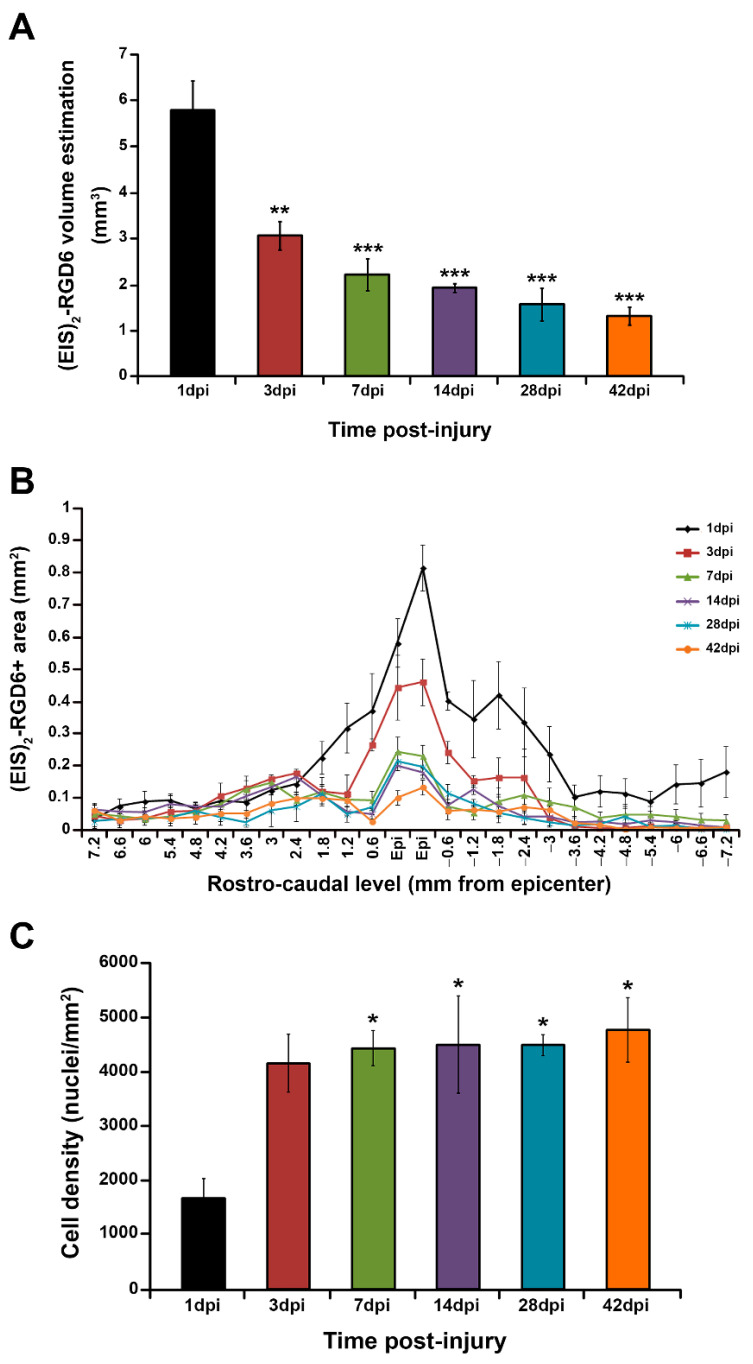
Quantitative analysis of (EIS)_2_-RGD6 degradation and cell infiltration. Graphs show data obtained from the quantification of biotinylated (EIS)_2_-RGD6 degradation and cell infiltration [experiment (EIS)_2_-RGD6 II] at 1, 3, 7, 14, 28, and 42 days post-injury (dpi): (**A**) data obtained from quantification of the temporal evolution of (EIS)_2_-RGD6 estimated total volume (**, *p* < 0.01 and ***, *p* < 0.001 vs. 1 dpi); (**B**) data obtained from the quantification of the temporal evolution of (EIS)_2_-RGD6+ area at each evaluated rostro-caudal level; and (**C**) data obtained from the temporal evolution of cell density in the (EIS)_2_-RGD6 porous scaffold in the lesion epicentre (*, *p* < 0.05 vs. 1 dpi). In (**A**,**C**), the potential existence of statistically significant between-group differences was assessed by one-way ANOVA followed by the Bonferroni post-hoc test.

In accordance with the previously described qualitative observations, quantification of (EIS)_2_-RGD6 biodegradability indicated that most (EIS)_2_-RGD6 degradation took place during the first 7 dpi, as approximately 60% of the observed (EIS)_2_-RGD6 at 1 dpi had disappeared at 7 dpi (Figure 13A). From 7 to 42 dpi, (EIS)_2_-RGD6 degradation was less evident, with the amount of (EIS)_2_-RGD6 observed at the end of the study (42 dpi) being approximately 20% of that observed at 1 dpi (Figure 13A). Furthermore, in accordance with the abovementioned qualitative observations, evaluation of the temporal degradation of (EIS)_2_-RGD6 at each rostro-caudal level demonstrated that (EIS)_2_-RGD6 disappearance was mainly observed in the lesion epicentre, at the adjacent evaluated spinal cord levels and, to a lesser extent, at rostral and caudal levels separated from the lesion site (Figure 13B).

#### 3.2.3. Quantification of (EIS)_2_-RGD6 Cell Infiltration

As previously stated, one of the most important features of hydrogel scaffolds to be used in SCI is that they should be favourable for cell adhesion and infiltration. In this regard, we found that, at all evaluated survival times, the cell nuclei were observed to be present inside the porous scaffold of (EIS)_2_-RGD6. Moreover, quantification of cell density in the (EIS)_2_-RGD6 porous scaffold during the temporal progression of SCI demonstrated a robust increase in this parameter from 1 to 7 dpi, while from 7 dpi cell density remained stable until 42 dpi (Figure 13C).

#### 3.2.4. Determination of (EIS)_2_-RGD6 Porosity

**Figure 14:**
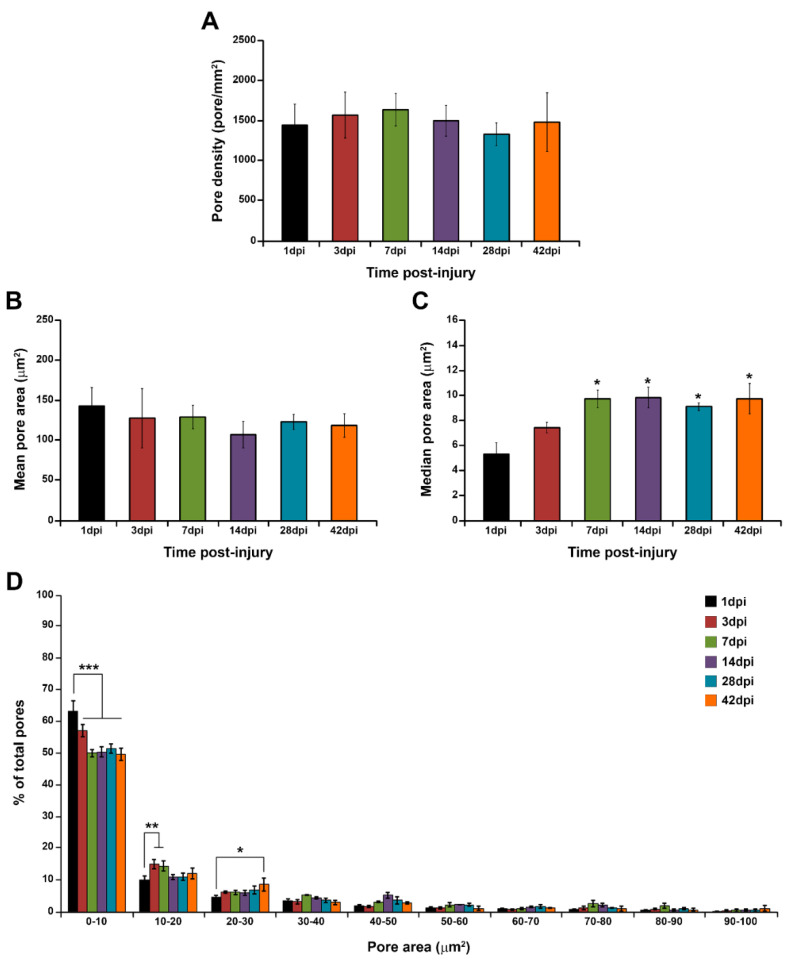
Quantitative analysis of (EIS)_2_-RGD6 porosity. Graphs show data obtained from the quantification of biotinylated (EIS)_2_-RGD6 porosity [experiment (EIS)_2_-RGD6 II] at 1, 3, 7, 14, 28, and 42 days post-injury (dpi) in the (EIS)_2_-RGD6 porous scaffold in the lesion epicentre: (**A**) data from the quantification of the temporal evolution of pore density; (**B**) data obtained from the quantification of the temporal evolution of the mean pore area; (**C**) data obtained from the quantification of the temporal evolution of the median pore area; and (**D**) data obtained from the quantification of the temporal evolution of pore size distribution (*, *p* < 0.05; **, *p* < 0.01; and ***, *p* < 0.001). In (**A**–**C**), the potential existence of statistically significant between-group differences was assessed by one-way ANOVA followed by the Bonferroni post-hoc test while, for (**D**), two-way ANOVA followed by the Bonferroni post-hoc test was conducted.

Quantification of the degree of porosity of the (EIS)_2_-RGD6 porous scaffold indicated no differences in pore density during the temporal progression of the injury (Figure 14A). Regarding pore size, at all evaluated times post-injury, the mean pore area was in the range of 110–150 µm^2^, without significant variations (Figure 14B); however, when the median pore area was evaluated, a significant increase in this parameter was observed from 1 to 7 dpi, while no differences were found from 7 to 42 dpi (Figure 14C). Finally, analysis of the pore size distribution showed a wide range of pore areas, although approximately 90% of pores displayed an area between 1 and 100 µm^2^ (Figure 14D). Moreover—and accordingly to that observed in the median pore size analysis—we found a slight augmentation in pore size with increasing time post-injury; although, in general, the pore size distribution remained highly stable over the evaluated time period (Figure 14D).

### 3.3. Effects of (EIS)_2_-RGD6 Injection after SCI

Finally, a third experimental set [experiment (EIS)_2_-RGD6 III] was carried out in order to corroborate the previously detailed data regarding motor functional recovery and white matter preservation at 42 dpi, as well as to assess white matter preservation at an intermediate survival time (7 dpi). Moreover, we aimed to analyse whether, at both 7 and 42 dpi, (EIS)_2_-RGD6 administration influences SCI-associated fibrosis and microglia/macrophage and astroglial reactivity, which are pivotal biological processes that not only greatly determine the progression and outcome of SCI, but also play an essential role in the tissue response to the presence of a foreign body (please note that, as mentioned above, a unique injection of 6 µL in the lesion epicentre was used for this experimental set).

#### 3.3.1. Evaluation of Motor Functional Recovery

**Figure 15:**
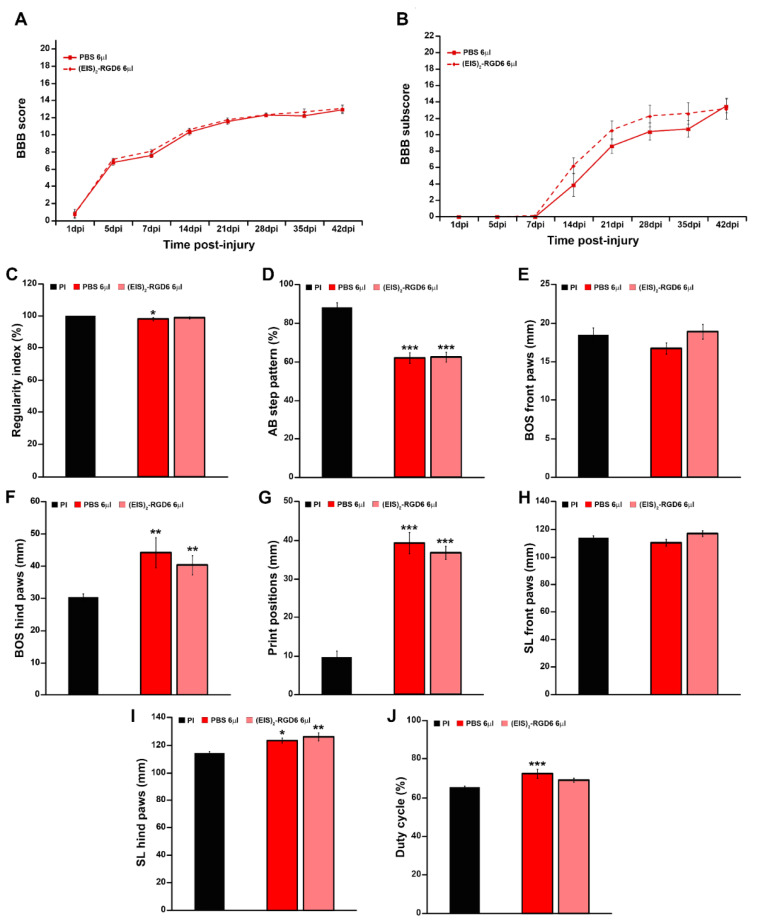
Evaluation of the effects exerted by (EIS)_2_-RGD6 injection in motor functional recovery. Graphs show data obtained from the evaluation of the effects exerted by (EIS)_2_-RGD6 injection in motor functional recovery in animals used to perform the experiment (EIS)_2_-RGD6 III. (**A**) Data from motor functional recovery assessment using the 21-point Basso, Beattie, and Bresnahan open-field test (BBB) score at 1, 5, 7, 14, 1, 28, 35, and 42 days post-injury (dpi); (**B**) data from motor functional recovery evaluation using the BBB sub-score at the same times post-injury; and (**C**–**J**) data from motor functional recovery assessment at 42 dpi using the Catwalk^®^ gait analysis system. The following gait parameters were evaluated: (**C**) regularity index [*, *p* < 0.05 vs. pre-injury (PI)]; (**D**) AB step pattern (***, *p* < 0.001 vs. PI); (**E**) front paws base of support (BOS); (**F**) hind paws BOS (**, *p* < 0.01 vs. PI); (**G**) print positions (***, *p* < 0.001 vs. PI); (**H**) front paws stride length (SL); (**I**) hind paws SL (*, *p* < 0.05 and **, *p* < 0.01 vs. PI); and (**J**) duty cycle (***, *p* < 0.001 vs. PI). In (**A**,**B**), the potential existence of statistically significant between-group differences was assessed by two-way ANOVA followed by the Bonferroni post-hoc test while, for (**C**–**J**), one-way ANOVA followed by the Bonferroni post-hoc test was conducted. Please note that data obtained from the separate analysis of the individual BBB parameters evaluated can be found in Table S3.

Similar to what was observed in experiment (EIS)_2_-RGD6 I, we did not observe statistically significant differences between the (EIS)_2_-RGD6 6 µL and PBS 6 µL groups in either the BBB score (Figure 15A), BBB sub-score (Figure 15B), or the different gait parameters evaluated using the Catwalk^®^ gait analysis system (Figure 15C–J), thereby corroborating that intraparenchymal injection of 6 µL of (EIS)_2_-RGD6 in the lesion epicentre immediately after SCI did not worsen motor functional recovery, when compared to its corresponding PBS-injected control group (data obtained from the separate analysis of the individual BBB parameters evaluated can be found in Table S3).

#### 3.3.2. Analysis of White Matter Preservation

**Figure 16:**
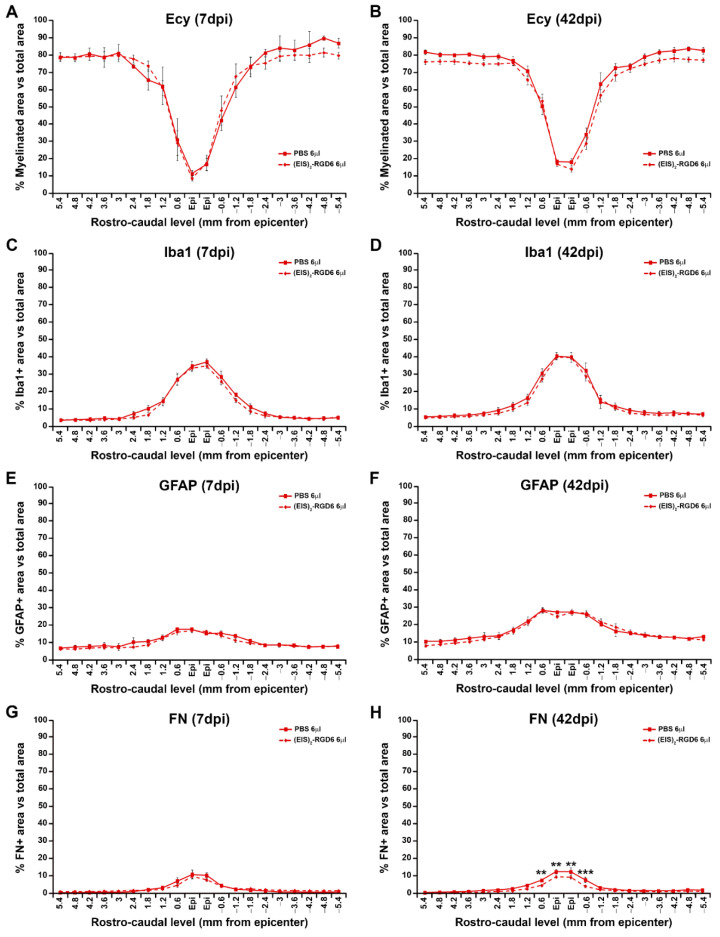
Analysis of the effects exerted by (EIS)_2_-RGD6 injection in myelin preservation, fibrosis, and microglia/macrophage and astroglial reactivity. Graphs show data obtained from the densitometric quantification of (**A**,**B**) myelin preservation [eriochrome cyanine (Ecy) staining]; (**C**,**D**) microglia/macrophage reactivity [ionized calcium-binding adaptor molecule 1 (Iba1) immunostaining]; (**E**,**F**) astroglial reactivity [glial fibrillary acidic protein (GFAP) immunostaining]; and (**G**,**H**) fibrosis [fibronectin (FN) immunostaining]. **, *p* < 0.01 and ***, *p* < 0.001 PBS 6 µL group vs. (EIS)_2_-RGD6 at 7 (**A**,**C**,**E**,**G**) and 42 days post-injury (dpi) (**B**,**D**,**F**,**H**) in animals used to perform experiment (EIS)_2_-RGD6 III. Please note that representative images from Ecy, Iba1, GFAP, and FN stained sections can be found in Figure 17, Figure 18, Figure 19 and Figure 20, respectively.

**Figure 17:**
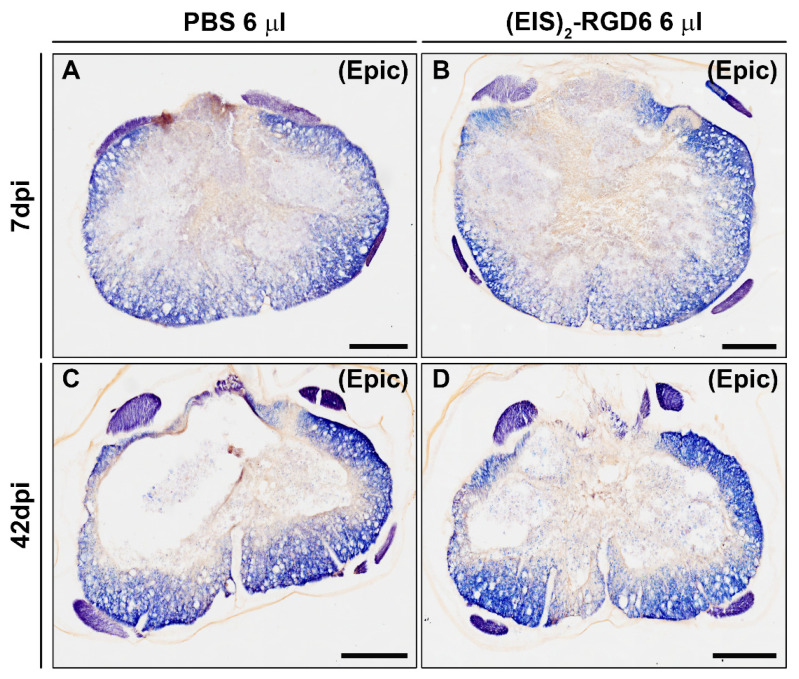
Representative images from eriochrome cyanine (Ecy) stained sections. Representative images of Ecy stained spinal cord sections corresponding to the lesion epicentre, used to carry out the densitometrical analysis of myelin preservation in experiment (EIS)_2_-RGD6 III at 7 and 42 days post-injury (dpi): (**A**) PBS 6 µL group at 7 dpi; (**B**) (EIS)_2_-RGD6 6 µL group at 7 dpi; (**C**) PBS 6 µL group at 42 dpi; and (**D**) (EIS)_2_-RGD6 6 µL group at 42 dpi. Scale bars, 500 µm. Please note that data obtained from the densitometric analysis of myelin preservation in these sections can be found in Figure 16A,B.

Analysis of Ecy stained sections again reinforced the observations from experiment (EIS)_2_-RGD6 I, as no significant between-group differences were detected in spared white matter at the end of the study (42 dpi); see Figure 16B. Moreover, no changes in this parameter were observed at 7 dpi (Figure 16A), supporting the conclusion that the injection of 6 µL of (EIS)_2_-RGD6 did not induce further damage than that observed in those animals that received an equivalent injection of PBS (representative images from Ecy stained sections are shown in Figure 17).

#### 3.3.3. Evaluation of Microglia/Macrophage and Astroglial Reactivity

**Figure 18:**
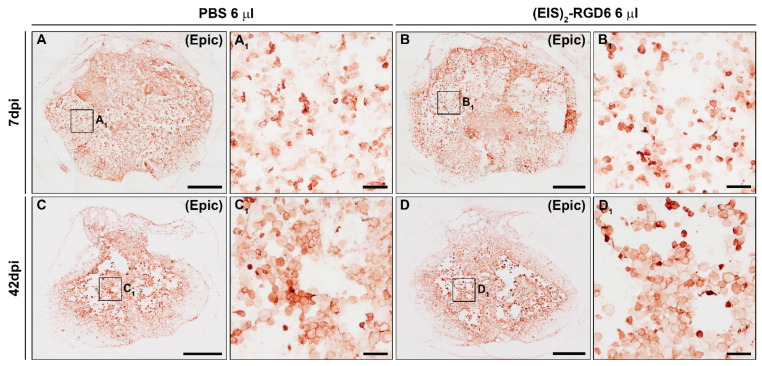
Representative images from ionized calcium-binding adaptor molecule 1 (Iba1) immunostained sections. Representative images of Iba1 immunostained spinal cord sections corresponding to the lesion epicentre, used to carry out the densitometrical analysis of microglia/macrophage reactivity in experiment (EIS)_2_-RGD6 III at 7 and 42 days post-injury (dpi): (**A**,**A_1_**), PBS 6 µL group at 7 dpi; (**B**,**B_1_**) (EIS)_2_-RGD6 6 µL group at 7 dpi; (**C**,**C_1_**), PBS 6 µL group at 42 dpi; and (**D**,**D_1_**) (EIS)_2_-RGD6 6 µL group at 42 dpi. Scale bars in (**A**–**D**), 500 µm. Scale bars in (**A_1_**–**D_1_**), 50 µm. Please note that data obtained from the densitometric analysis of microglia/macrophage reactivity in these sections can be found in Figure 16C,D.

**Figure 19:**
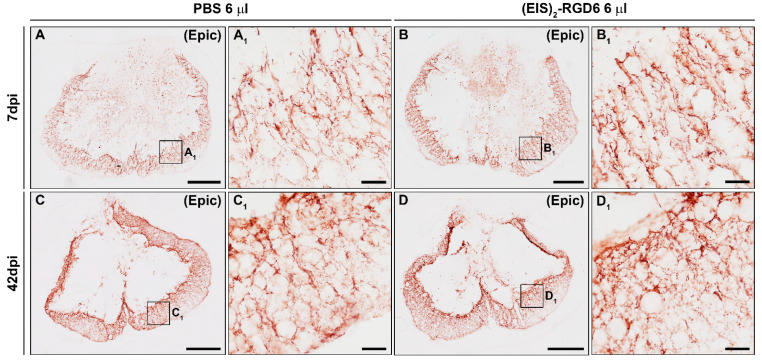
Representative images from glial fibrillary acidic protein (GFAP) immunostained sections. Representative images of GFAP immunostained spinal cord sections corresponding to the lesion epicentre, used to carry out the densitometrical analysis of astroglial reactivity in experiment (EIS)_2_-RGD6 III at 7 and 42 days post-injury (dpi): (**A**,**A_1_**) PBS 6 µL group at 7 dpi; (**B**,**B_1_**) (EIS)_2_-RGD6 6 µL group at 7 dpi; (**C**,**C_1_**) PBS 6 µL group at 42 dpi; and (**D**,**D_1_**) (EIS)_2_-RGD6 6 µL group at 42 dpi. Scale bars in (**A**–**D**), 500 µm. Scale bars in (**A_1_**–**D_1_**), 50 µm. Please note that data obtained from the densitometric analysis of astroglial reactivity in these sections can be found in Figure 16E,F.

Densitometric quantification of microglia/macrophage and astroglial reactivity demonstrated that (EIS)_2_-RGD6 injection did not influence these biological processes after SCI, as no differences were found in the presence of activated microglia/macrophages (Figure 16C,D) and astroglial cells (Figure 16E,F) in the lesioned areas at either 7 dpi or 42 dpi (representative images of spinal cord sections processed for the visualization of Iba1 and GFAP can be found in Figure 18 and Figure 19, respectively); accordingly, no qualitative morphological changes were observed in activated microglia/macrophages (Figure 18) and astroglial cells (Figure 19) at any of the evaluated survival times. Altogether, these observations strongly suggest that intraparenchymal (EIS)_2_-RGD6 injection did not further increase the SCI-associated inflammatory response and glial scarring.

#### 3.3.4. Assessment of Fibrosis

**Figure 20:**
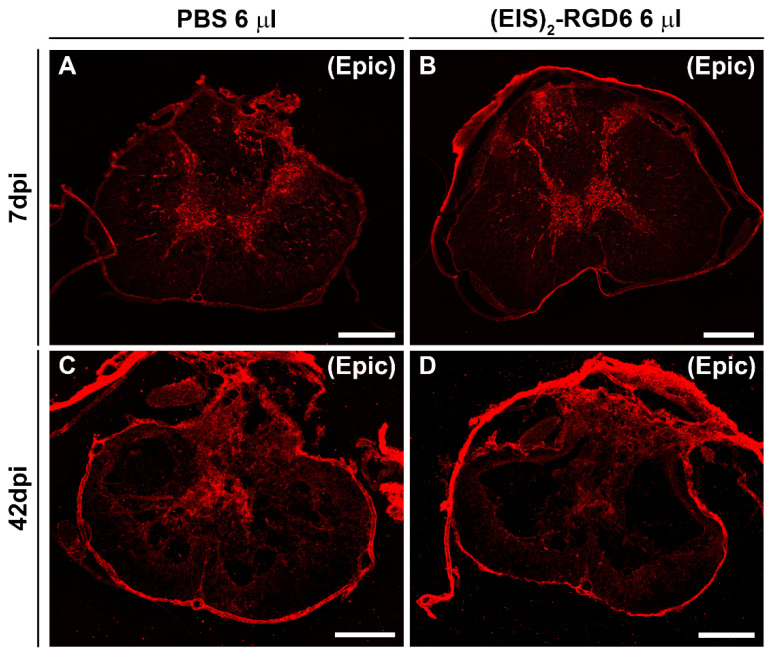
Representative images from fibronectin (FN) immunostained sections. Representative images from FN immunostained spinal cord sections corresponding to the lesion epicentre, used to carry out the densitometric analysis of fibrosis in experiment (EIS)_2_-RGD6 III at 7 and 42 days post-injury (dpi): (**A**) PBS 6 µL group at 7 dpi; (**B**) (EIS)_2_-RGD6 6 µL group at 7 dpi; (**C**) PBS 6 µL group at 42 dpi; and (**D**) (EIS)_2_-RGD6 6 µL group at 42 dpi. Scale bars, 500 µm. Please note that data obtained from the densitometric analysis of fibrosis in these sections can be found in Figure 16G,H.

In contrast to the lack of (EIS)_2_-RGD6-mediated effects on microglial and astroglial reactivity, we found that, at the end of the study (42 dpi), when compared to the PBS-injected control group, animals that were subjected to (EIS)_2_-RGD6 injection displayed statistically significant lower fibrosis in the lesion epicentre at and the adjacent rostro–caudal spinal cord levels (Figure 16H). No between-group differences were observed in this parameter at 7 dpi (Figure 16g). Representative images from spinal cord sections processed for the visualization of FN can be found in Figure 20.

## 4. Discussion

As previously stated, a great amount of effort has been put into the search for new biomaterials that could be used to develop suitable treatments for SCI. Despite the outstanding physicochemical and biological properties of SELPs, to date, no currently available information on the potential use of SELPs in SCI can be found in the scientific literature. In this context, the present study represents the first experimental approach aiming to evaluate the potential usefulness of SELPs in this neuropathological condition.

As a first essential point of discussion, it should be noted that one of the most important aspects in the design of hydrogel-based approaches for SCI treatment is the timing [63,64], as hydrogel implantation during the acute phase of SCI (even in the case of minimally invasive intraparenchymal injection of in situ-gelling hydrogels) might cause further damage in the lesioned spinal cord tissue by increasing intraspinal pressure, due to the injection process or hydrogel swelling [65]. Although it is usually assumed that the intraparenchymal injection of large volumes during the acute phase of SCI will induce further spinal cord tissue injury, to the best of our knowledge there are no currently available studies that have systematically and quantitatively determined the range of acceptable injection volumes for this neuropathological condition. Moreover—and given that the potential deleterious effects derived from intraparenchymal injection in the lesioned spinal cord largely depend on a plethora of specific experimental and/or technical aspects [66]—it is critical to determine the optimal injection system in each particular experimental situation [65]. In the present study, we found that, in all tested injection systems, PBS injection worsened motor functional recovery during SCI progression at specific times post-injury, although no differences were found in myelin preservation at the end of the study. However, we observed an evident relationship between increased PBS volumes and/or injection points and more pronounced functional impairment, with the group that received a single injection of 6 µL in the lesion site being the one that displayed the most similar motor functional recovery to that observed in NI rats, as well as showing an almost indistinguishable motor performance at the end of the study. Moreover, it also should be noted that intraparenchymal injection of (EIS)_2_-RGD6 did not impair motor functional recovery and myelin preservation, when compared to that observed in the corresponding PBS-injected control groups, thereby supporting the conclusion that (EIS)_2_-RGD6 swelling did not induce further damage in the lesioned spinal cord. In accordance, it has been previously reported that protein-based hydrogels—such as those used in the present study—tend to swell minimally [65]. Furthermore, the lack of negative impact observed in (EIS)_2_-RGD6-injected animals, when compared to corresponding PBS-injected controls, also suggested that (EIS)_2_-RGD6 presents similar mechanical characteristics to those displayed by the spinal cord tissue, as a mismatch between the mechanical properties of a biomaterial and the lesioned spinal cord generally lead to affectation of the surrounding tissue and, thus, worsened SCI outcome [67]. Although we do not know the exact stiffness of (EIS)_2_-RGD6 at the specific concentration used in the present study (50 mg/mL), previous reports have indicated that, at a concentration of 100 mg/mL in PBS, this SELP displays a modulus of approximately 150 Pa at physiological temperature [51,52]. On the other hand, despite the existence of discrepancies in the mechanical properties of the spinal cord [67], there is a tendency to consider that an elastic modulus between 3–300 kPa could be the acceptable range for a biomaterial to be used in the lesioned spinal cord [13,17,68]; meanwhile, interestingly, a growing number of reports have shown that softer hydrogels are more permissive for neurite growth and tissue regeneration [69,70,71,72,73], even in the case of ELP scaffolds [74]. Taken together, these observations point to the suitability of the use of (EIS)_2_-RGD6, under optimized conditions, regarding its intraparenchymal injection during the acute phase after SCI, without inducing further damage. This is a major advantage, as many of the deleterious biological processes that lead to the affectation of the initially uninjured spinal cord tissue take place during the initial phase of SCI progression and, thereby, great efforts have been made to unravel therapeutic approaches for early intervention after SCI [75,76,77].

Regarding the distribution of (EIS)_2_-RGD6—and also as a major advantage displayed by this SELP in the lesioned spinal cord—our results indicated that a single injection of 6 µL of (EIS)_2_-RGD6 in the lesion epicentre led to an excellent widespread distribution of this SELP, covering all the lesioned areas, being mainly observed in the lesion epicentre and at adjacent rostro–caudal spinal cord levels, as well as in the dorsal columns and the grey matter surrounding them at rostro–caudal spinal cord levels separated from the injury site, which are thought to be affected in the secondary progression of SCI. Interestingly, most of the few studies that have assessed the in vivo injectable hydrogel distribution after its intraparenchymal administration during the acute phase of SCI in clinically relevant animal models of spinal cord contusion and/or compression have shown that the hydrogel distribution was generally confined to the lesion epicentre [78,79,80,81,82], suggesting that its potential beneficial effects and those derived from the therapeutic drugs or cells that could be combined with them would likely be limited to these areas. However, spinal cord tissue from more separated rostro–caudal spinal cord levels is also affected due to the secondary injury associated to the progression of SCI and, therefore, also requires therapeutic intervention and, thus, the development of effective hydrogel-based SCI therapeutic approaches.

Moreover, it is interesting to note that two different (EIS)_2_-RGD6 structures were clearly observed, depending on the distance to the injection point (lesion site). On one hand, a porous scaffold was found in the lesion epicentre and at adjacent rostro–caudal spinal cord levels, where a cystic cavity is formed during the temporal progression of contusive SCI [83] and, thereby, a hydrogel bridge is required to create a more permissive and supportive environment to promote tissue regeneration, revascularization, and axonal growth, as well as to overcome the main drawbacks of classic cell transplantation and drug delivery strategies, by increasing the viability and retention in the lesion site of those cell transplants and/or molecules that could be combined with it [6]. On the other hand, at those rostro–caudal spinal cord levels separated from the injury epicentre, (EIS)_2_-RGD6 was structured as spared fibres and/or particles in the injured spinal cord tissue. Notably, in these lesioned regions, a cystic cavity is not usually formed and, therefore, the formation of a porous hydrogel scaffold is not needed to bridge it. However, and as mentioned above, these areas are also affected due to the secondary progression of SCI and thus would benefit from the potential beneficial effects of the hydrogel and its potential therapeutic combinations. Otherwise, another pivotal aspect of the hydrogel scaffold structure to be used in SCI is its porosity and pore size which, as previously detailed, critically determines the diffusion of soluble factors such as nutrients, ions, waste products, and potential therapeutic compounds, as well as the infiltration, proliferation, and functioning of endogenous cells to promote tissue regeneration [84]. Despite the key role of porosity and pore size in the effects exerted by biomaterials, it is surprising that, to date, there are no published studies that have systematically assessed the effects of biomaterial porosity and pore size on spinal cord regeneration. Nevertheless, from the reports that have partially and/or indirectly evaluated the influence of porosity and pore size on the infiltration, proliferation, and function of neural cells in biomaterial scaffolds, it can be extrapolated that hydrogels to be used in SCI should display the highest possible porosity without compromising mechanical strength [85] and that the optimal pore size seems to vary depending on the specific cell type and/or biological process [10,15]; for instance, axons show a preference for small pores to regenerate, while large pores are needed for proper neovascularization [84,86,87]. Consequently, the use of scaffolds displaying different pore sizes would be desirable in hydrogel-based strategies for tissue regeneration [15,84]. In this regard, we showed that acute intraparenchymal (EIS)_2_-RGD6 injection in the lesioned spinal cord led to the formation of a porous scaffold in the lesion epicentre displaying an ample range of pore sizes, as has been previously described in vitro [51]. More importantly, we found that the cell density in the (EIS)_2_-RGD6 scaffold significantly increased with the temporal progression of SCI, demonstrating that its porosity and pore size allow for endogenous cell infiltration and/or proliferation, in accordance with previous in vitro observations [49]. Future studies will be needed to ascertain the specific cell types that infiltrate and/or proliferate in the (EIS)_2_-RGD scaffold, as well as to determine whether axonal growth and neovascularization occur, as has been described for other elastin-based biomaterials [27,29,30,31,32].

Another essential aspect of hydrogel-based approaches for SCI is the biodegradability of the biomaterial used since, as previously described, those biomaterials to be used in the lesioned spinal cord should be degradable in order to avoid the need for a surgical process to remove the scaffold [17,88]. Although the optimal timing of degradation has not been specifically determined in the lesioned spinal cord, there is currently consensus that, in order to achieve effective spinal cord repair, the degradation rate of the biomaterial should be slow enough to maintain its presence in the lesion site during the time period when tissue and axonal regeneration take place [5,10,17,89,90,91]. More specifically, and based on the rate of axonal regeneration in the corticospinal tract, it has been proposed that a degradation rate in the order of weeks to months would be appropriate in rodents [89]. In this context, it should be noted that one of the major disadvantages of biodegradable hydrogels is that, in many cases, they degrade too quickly to efficiently support long-term tissue and axonal regeneration [10,17,89,92,93,94]. This is not only of utmost importance for the promotion of endogenous spinal cord regeneration, but also for the potential combination of hydrogels with drugs or cell transplants that need to maintain their bioactivity and viability in the lesion site for longer periods to exert their beneficial roles. In the present study, we found that (EIS)_2_-RGD6 displayed long-term stability and a slow degradation rate, as the presence of the (EIS)_2_-RGD6 scaffold in the lesion site was still observed at the end of the study (42 dpi). Accordingly, previous reports have shown that this SELP is able to form scaffolds with high long-term stability both in vitro and in vivo after subcutaneous injection [49,51], likely due to the fact that (EIS)_2_-RGD6 does not present specific sites for enzymatic recognition in its amino acid sequence. Moreover, an ideal scaffold for SCI regeneration should maintain its mechanical strength throughout the regenerative and degradation process, as the loss of mechanical integrity during hydrogel degradation may lead to the collapse of the scaffold and, thereby, to the failure of tissue regeneration [12,95]. As previously reported [96], biomaterial degradation generally occurs through surface or bulk erosion, depending on whether degradation takes place mainly at the external surface of the scaffold or homogeneously (i.e., with exterior and interior bonds being degraded simultaneously). Interestingly, bulk erosion might lead to the collapse of the scaffold architecture due to the loss of its mechanical strength while, in contrast, surface erosion may allow for maintenance of the structural stability of the scaffold during the degradation process [12]. In this line, we observed that the pore size of the (EIS)_2_-RGD6 scaffold during its degradation remained very stable, strongly supporting the conclusion that surface and not bulk erosion underlies (EIS)_2_-RGD6 scaffold degradation. Accordingly, no signs of scaffold collapse were observed during the assessed time period, thereby supporting the conclusion that the (EIS)_2_-RGD6 scaffold is able to maintain its structural stability during its degradation process in the lesioned spinal cord.

Importantly, different reports have shown that non-degradable and slow-degrading hydrogels, such as the one used in the present study, seem to display a higher capacity to induce an inflammatory foreign body response in the lesioned spinal cord, when compared to rapidly-degrading hydrogels [6,97]. The foreign body response has been defined as an inflammatory-mediated reaction of the tissue to implanted biomaterials, which encompasses a wide range of inter-related cellular and molecular processes that induce chronic inflammation, scarring, and encapsulation of the implant, thereby leading to a lack of tissue regeneration [98]. As a consequence, an enormous amount of effort has been put into unravelling new biomaterials and/or biomaterials-based approaches that do not induce (or, even, reduce) such a deleterious tissue response [99,100,101]. Moreover, the induction of the foreign body response is of special importance in the context of SCI since, as has been widely described [2,102], the progression of this neuropathological condition is accompanied by a prominent inflammatory response which, together with the formation of a glial and fibrous scar, greatly influences the histopathological and functional outcome. Among the different cell types involved in the inflammatory foreign body response in the lesioned spinal cord, activated microglia/macrophages, astroglial cells, and fibroblasts are critical players, due to their pivotal role in the SCI-associated inflammatory response and the formation of glial and fibrous scars [103,104,105,106,107,108]. In this regard, the results obtained in the present work demonstrate that, despite its slow-degrading property, the presence of (EIS)_2_-RGD6 in the lesioned spinal cord did not induce further microglia/macrophage reactivity and astroglial scarring than that observed due to the injury at any of the evaluated time-points. Moreover, we also found that the presence of this SELP led to a significant reduction in fibrosis in the lesion epicentre which, based on previous reports demonstrating the deleterious effect of fibrotic scarring after SCI [109] and the critical role of fibrosis in the foreign body response [98], should be considered a major advantage of the use of (EIS)_2_-RGD6 in the lesioned spinal cord. In addition, all of these observations strongly support the fact that the by-products derived from its degradation are not immunogenic, in accordance with previous reports showing that elastin-derived peptides did not induce an inflammatory response in primary astroglial cultures [44].

In conclusion, as a first experimental approach on the usefulness of SELPs in SCI, we herein demonstrated, in a clinically relevant SCI model, that the injectable in situ-gelling SELP named (EIS)_2_-RGD6 is a promising polymer for the design of hydrogel-based strategies for the treatment of SCI. Under optimized conditions, it can be acutely injected in the lesioned spinal cord without inducing further damage, showing an excellent distribution covering all lesioned areas with a single injection and forming a slow-degrading porous scaffold in the lesion site that allows for the infiltration and/or proliferation of endogenous cells with no signs of collapse. Meanwhile, it presented inert effects on microglial and astroglial reactivity and glial scarring, while remarkably reducing SCI-associated fibrosis. We hope that the results obtained in the present study—together with the need for novel biomaterials to be used in SCI and the previously described outstanding design flexibility, control over polymer composition, monodispersity, tailorability, and combinability with potential therapeutic drugs and/or cell transplants displayed by this type of biomaterial—will open new and interesting research avenues focused on the development of novel and effective SELP biomaterial-based strategies for the treatment of this devastating neuropathological condition.

## Data Availability

Data are contained within the article or Supplementary Materials.

## Supplementary Materials

The following supporting information can be downloaded at: https://www.mdpi.com/article/10.3390/pharmaceutics14122713/s1. Figure S1: Representative images from eriochrome cyanine stained sections; Figure S2: Qualitative microscopic analysis of (EIS)_2_-RGD6 distribution at 3 days post-injury; Figure S3: Qualitative microscopic analysis of (EIS)_2_-RGD6 distribution at 7 days post-injury; Figure S4: Qualitative microscopic analysis of (EIS)_2_-RGD6 distribution at 14 days post-injury; Figure S5: Qualitative microscopic analysis of (EIS)_2_-RGD6 distribution at 28 days post-injury; Figure S6: Qualitative microscopic analysis of (EIS)_2_-RGD6 structure at 3 days post-injury; Figure S7: Qualitative microscopic analysis of (EIS)_2_-RGD6 structure at 7 days post-injury; Figure S8: Qualitative microscopic analysis of (EIS)_2_-RGD6 structure at 14 days post-injury; Figure S9: Qualitative microscopic analysis of (EIS)_2_-RGD6 structure at 28 days post-injury; Table S1; Separate analysis of 21-point Basso, Beattie, and Bresnahan open-field test (BBB) individual aspects; Table S2: Contingency tables from mortality rate evaluation; Table S3: Separate analysis of 21-point Basso, Beattie, and Bresnahan open-field test (BBB) individual aspects.
