# Supplementary material for: Silk-Elastin-like Polymers for Acute Intraparenchymal Treatment of the Traumatically Injured Spinal Cord: A First Systematic Experimental Approach
Source: Pharmaceutics. 2022 Dec 3;14(12):2713. doi: 10.3390/pharmaceutics14122713 (PMC9784492; doi:10.3390/pharmaceutics14122713)
Supplement: Supplementary file 1 [file pharmaceutics-14-02713-s001.zip › Figure S1.pdf]

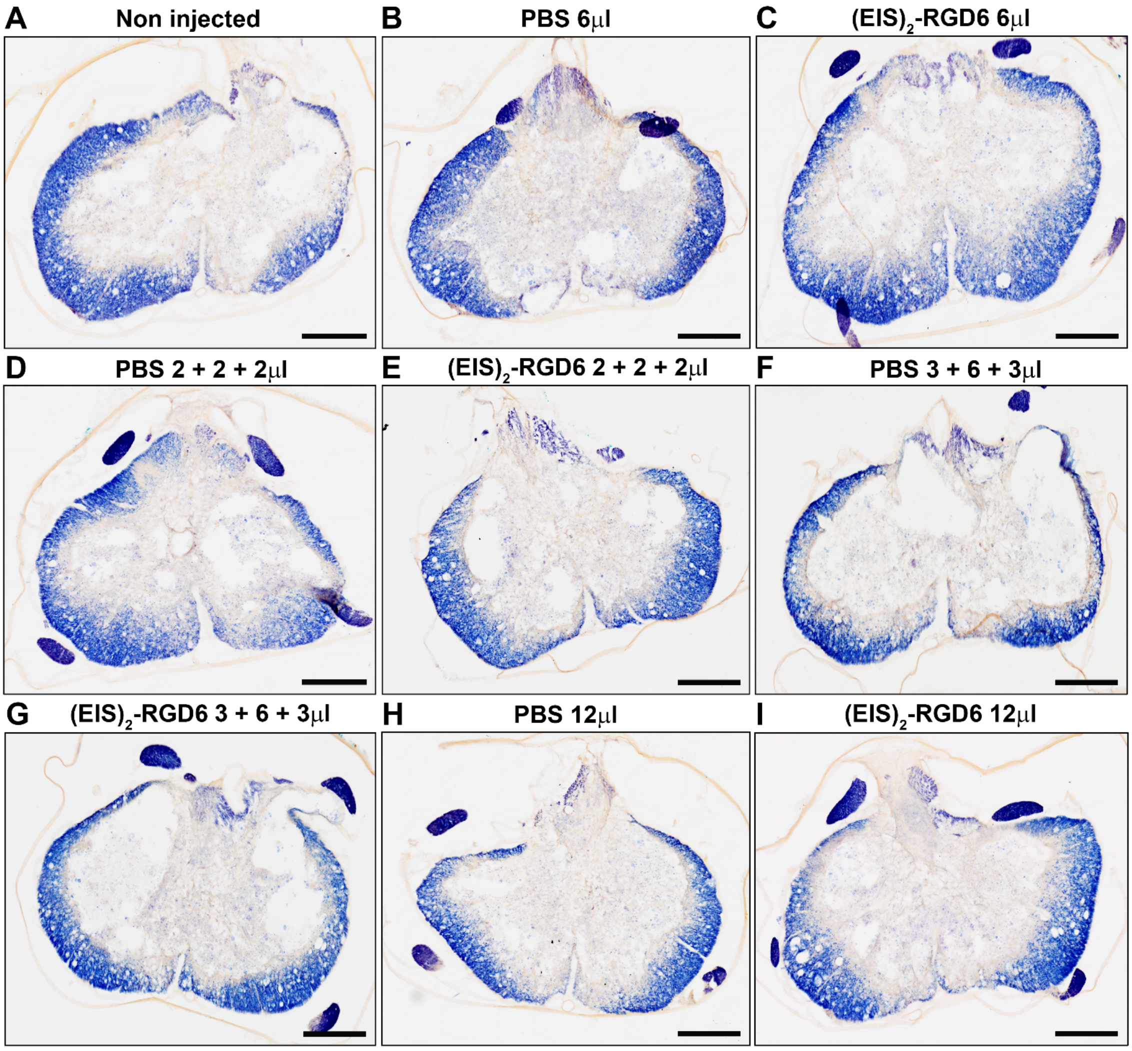

Figure S1. Representative images from eriochrome cyanine stained sections. Representative images of Ecy stained spinal cord sections corresponding to the lesion epicentre, used to carry out the densitometrical analysis of myelin preservation in experiment $(EIS)_2$-RGD6 I: (A) Non-injected group; (B) PBS 6 μL group; (C) $(EIS)_2$-RGD6 6 μL group; (D) PBS 2 + 2 + 2 μL group; (E) $(EIS)_2$-RGD6 2 + 2 + 2 μL group; (F) PBS 3 + 6 + 3 μL group; (G) $(EIS)_2$-RGD6 3 + 6 + 3 μL group; (H) PBS 12 μL group; and (I) $(EIS)_2$-RGD6 12 μL group. Scale bars, 500 μm. Please note that data obtained from the densitometric analysis of myelin preservation in these sections can be found in Figure 7.
