# Supplementary material for: Silk-Elastin-like Polymers for Acute Intraparenchymal Treatment of the Traumatically Injured Spinal Cord: A First Systematic Experimental Approach
Source: Pharmaceutics. 2022 Dec 3;14(12):2713. doi: 10.3390/pharmaceutics14122713 (PMC9784492; doi:10.3390/pharmaceutics14122713)
Supplement: Supplementary file 1 [file pharmaceutics-14-02713-s001.zip › Figure S3.pdf]

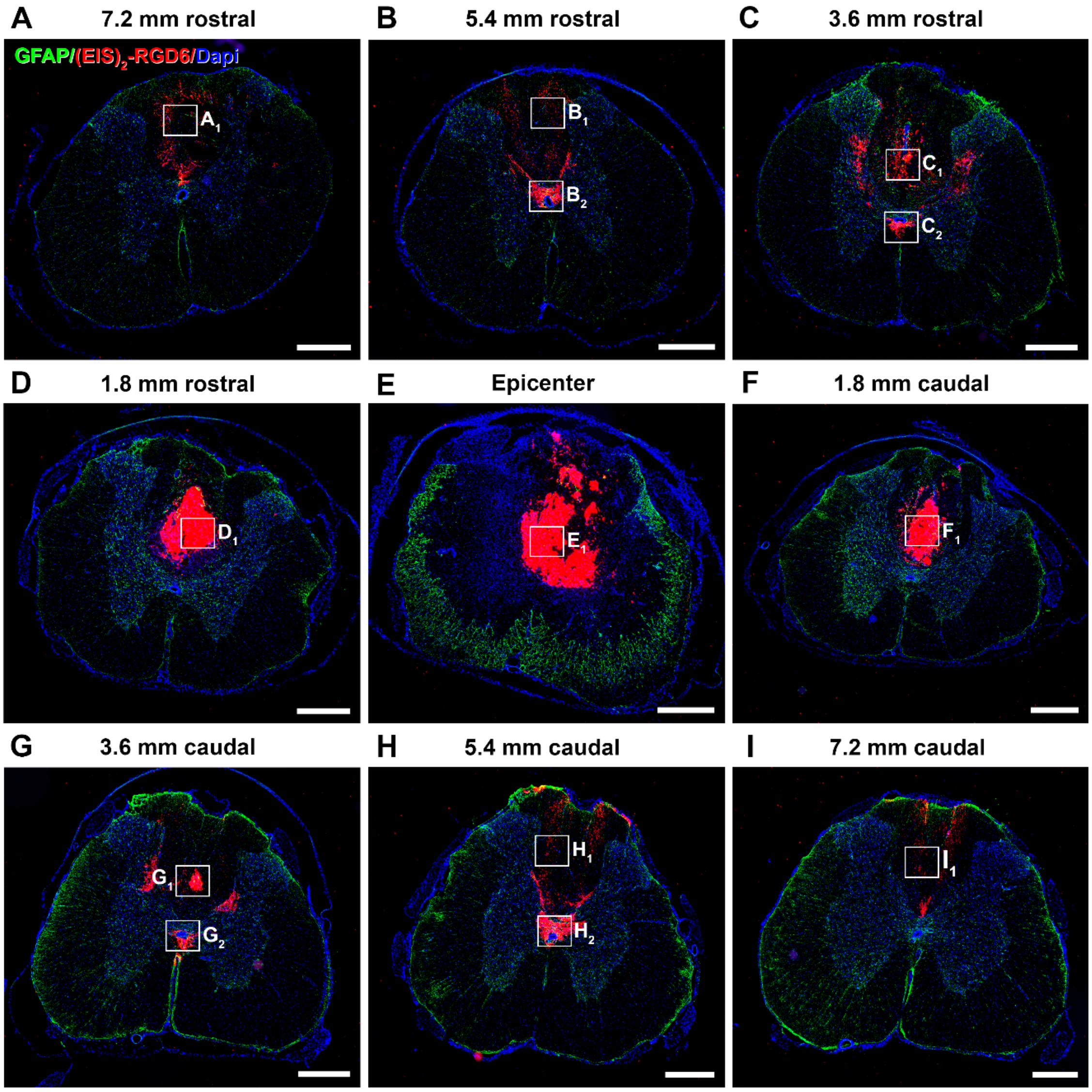

Figure S3. Qualitative microscopic analysis of (EIS)2-RGD6 distribution at 7 days post-injury. Representative images of whole spinal cord sections processed for the visualization of biotinylated (EIS)2-RGD6 and glial fibrillary acidic protein (GFAP) at 7 days post-injury in experiment (EIS)2-RGD6 II. The following rostro-caudal spinal cord levels are shown: (A) 7.2 mm rostral; (B) 5.4 mm rostral; (C) 3.6 mm rostral; (D) 1.8 mm rostral; (E) 0 mm; (F) 1.8 mm caudal; (G) 3.6 mm caudal; (H) 5.4 mm caudal; and (I) 7.2 mm caudal from epicentre. Scale bars, 500 μm. Please note that higher magnification images corresponding to the areas highlighted with squares in (A–I) can be found in Figure S7.
