# Supplementary material for: Silk-Elastin-like Polymers for Acute Intraparenchymal Treatment of the Traumatically Injured Spinal Cord: A First Systematic Experimental Approach
Source: Pharmaceutics. 2022 Dec 3;14(12):2713. doi: 10.3390/pharmaceutics14122713 (PMC9784492; doi:10.3390/pharmaceutics14122713)
Supplement: Supplementary file 1 [file pharmaceutics-14-02713-s001.zip › Figure S7.pdf]

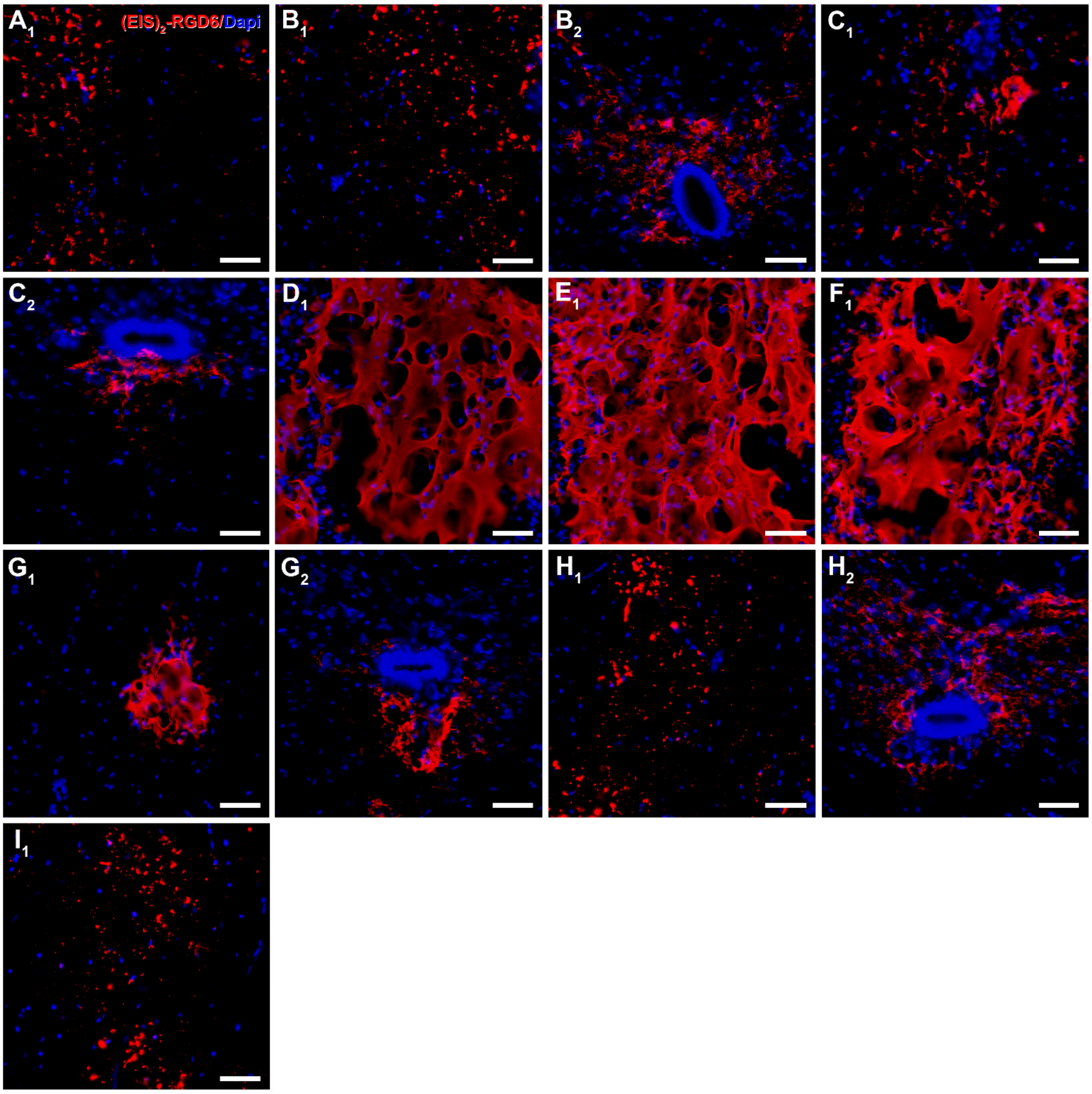

Figure S7. Qualitative microscopic analysis of (EIS)$_2$-RGD6 structure at 7 days post-injury. Representative higher magnification images of spinal cord sections processed for the visualization of biotinylated (EIS)$_2$-RGD6 at 7 days post-injury in experiment (EIS)$_2$-RGD6 II, showing (EIS)$_2$-RGD6 structure in the following representative rostro-caudal spinal cord levels: (A1) 7.2 mm rostral; (B1, B2) 5.4 mm rostral; (C1, C2) 3.6 mm rostral; (D1) 1.8 mm rostral; (E1) 0 mm; (F1) 1.8 mm caudal; (G1, G2), 3.6 mm caudal; (H1, H2), 5.4 mm caudal; and (I1) 7.2 mm caudal from the injury epicentre. Scale bars, 50 μm. Please note that the specific areas shown in these images are highlighted in Figure S3.
