# Supplementary material for: Silk-Elastin-like Polymers for Acute Intraparenchymal Treatment of the Traumatically Injured Spinal Cord: A First Systematic Experimental Approach
Source: Pharmaceutics. 2022 Dec 3;14(12):2713. doi: 10.3390/pharmaceutics14122713 (PMC9784492; doi:10.3390/pharmaceutics14122713)
Supplement: Supplementary file 1 [file pharmaceutics-14-02713-s001.zip › Table S1.pdf]

A. Non injected (NI) group vs. groups injected with PBS:

Paw positions

	1 dpi	5 dpi	7 dpi	14 dpi	21 dpi	28 dpi	35 dpi	42 dpi
**NI**	0 ± 0	0 ± 0	0 ± 0	1.25 ± 0.63	2.25 ± 0.85	2.5 ± 0.87	2.5 ± 0.65	2.25 ± 0.75
**PBS 6µl**	0 ± 0	0 ± 0	0 ± 0	0.5 ± 0.29	1.5 ± 0.29	1.5 ± 0.5	2 ± 0.41	2.75 ± 0.35
**PBS 2 + 2 + 2µl**	0 ± 0	0 ± 0	0 ± 0	0.4 ± 0.4	0.8 ± 0.37	1.6 ± 0.68	1.4 ± 0.68	1.6 ± 0.6
**PBS 3 + 6 + 3µl**	0 ± 0	0 ± 0	0 ± 0	0.2 ± 0.2	1 ± 0.32	1 ± 0.32	1.2 ± 0.2	1.6 ± 0.24
**PBS 12µl**	0 ± 0	0 ± 0	0 ± 0	0 ± 0	0.75 ± 0.75	0.5 ± 0.5	0.75 ± 0.48	0.5 ± 0.5

Toe clearance

	1 dpi	5 dpi	7 dpi	14 dpi	21 dpi	28 dpi	35 dpi	42 dpi
**NI**	0 ± 0	0 ± 0	0 ± 0	2 ± 0.71	4.25 ± 1.18	4.5 ± 0.5	4.25 ± 0.48	4.25 ± 0.48
**PBS 6µl**	0 ± 0	0 ± 0	0 ± 0	0.5 ± 0.5	2.25* ± 0.63	2.25* ± 0.63	3 ± 0.41	3 ± 0.41
**PBS 2 + 2 + 2µl**	0 ± 0	0 ± 0	0 ± 0	0.8 ± 0.58	1.2** ± 0.8	1.8** ± 0.66	2.4 ± 0.4	3.2 ± 0.8
**PBS 3 + 6 + 3µl**	0 ± 0	0 ± 0	0 ± 0	0.2* ± 0.2	0.6*** ± 0.4	1.4*** ± 0.24	1.6*** ± 0.24	1.8*** ± 0.37
**PBS 12µl**	0 ± 0	0 ± 0	0 ± 0	0* ± 0	0.5*** ± 0.5	0.5*** ± 0.5	0.75*** ± 0.47	0.5*** ± 0.5

Stepping

	1 dpi	5 dpi	7 dpi	14 dpi	21 dpi	28 dpi	35 dpi	42 dpi
**NI**	0 ± 0	0 ± 0	2 ± 0.7	6 ± 0	6 ± 0	6 ± 0	6 ± 0	6 ± 0
**PBS 6µl**	0 ± 0	0 ± 0	0*** ± 0	4.5** ± 0.96	6 ± 0	6 ± 0	6 ± 0	6 ± 0
**PBS 2 + 2 + 2µl**	0 ± 0	0 ± 0	0.5* ± 0.34	4*** ± 2	5.6 ± 0.4	6 ± 0	6 ± 0	6 ± 0
**PBS 3 + 6 + 3µl**	0 ± 0	0 ± 0	0*** ± 0	4.8* ± 0.73	6 ± 0	6 ± 0	6 ± 0	6 ± 0
**PBS 12µl**	0 ± 0	0 ± 0	0 ± 0	2.75** ± 1.25	2.75** ± 1.25	5 ± 1	5.75 ± 0.25	5 ± 1

Tail position

	1 dpi	5 dpi	7 dpi	14 dpi	21 dpi	28 dpi	35 dpi	42 dpi
**NI**	0 ± 0	0 ± 0	0 ± 0	0.25 ± 0.25	0.75 ± 0.25	1 ± 0.41	0.75 ± 0.25	0.75 ± 0.25
**PBS 6µl**	0 ± 0	0 ± 0	0 ± 0	0.5 ± 0.29	1 ± 0.41	1.25 ± 0.48	1.25 ± 0.25	1.25 ± 0.25
**PBS 2 + 2 + 2µl**	0 ± 0	0 ± 0	0 ± 0	0.6 ± 0.4	0.8 ± 0.2	1 ± 0.32	1.2 ± 0.37	1.2 ± 0.2
**PBS 3 + 6 + 3µl**	0 ± 0	0 ± 0	0 ± 0	0 ± 0	0.6 ± 0.24	0.6 ± 0.24	0.8 ± 0.37	0.8 ± 0.37
**PBS 12µl**	0 ± 0	0 ± 0	0 ± 0	0 ± 0	0* ± 0	0** ± 0	0.25 ± 0.25	0.75 ± 0.25

Coordination

	1 dpi	5 dpi	7 dpi	14 dpi	21 dpi	28 dpi	35 dpi	42 dpi
**NI**	0 ± 0	0 ± 0	0 ± 0	0.75 ± 0.48	1.75 ± 0.48	1.75 ± 0.48	2 ± 0.4	2.25 ± 0.48
**PBS 6µl**	0 ± 0	0 ± 0	0 ± 0	0 ± 0	0*** ± 0	0.75 ± 0.25	1.5 ± 0.29	2 ± 0.41
**PBS 2 + 2 + 2µl**	0 ± 0	0 ± 0	0 ± 0	0 ± 0	0*** ± 0	0.2*** ± 0.2	0.8** ± 0.2	1.4 ± 0.24
**PBS 3 + 6 + 3µl**	0 ± 0	0 ± 0	0 ± 0	0 ± 0	0*** ± 0	0.2*** ± 0.2	0.8* ± 0.2	1** ± 0.32
**PBS 12µl**	0 ± 0	0 ± 0	0 ± 0	0 ± 0	0*** ± 0	0.25** ± 0.25	0.75* ± 0.25	0.75** ± 0.25

B. PBS 6μl vs. (EIS)$_2$-RGD6 6μl:

Paw positions

	1 dpi	5 dpi	7 dpi	14 dpi	21 dpi	28 dpi	35 dpi	42 dpi
**PBS 6μl**	0 ± 0	0 ± 0	0 ± 0	0.5 ± 0.29	1.5 ± 0.29	1.5 ± 0.5	2 ± 0.41	2.75 ± 0.35
**(EIS)$_2$-RGD6 6μl**	0 ± 0	0 ± 0	0 ± 0	0.75 ± 0.5	1.5 ± 0.5	2.33 ± 0.33	2.67 ± 0.33	2.33 ± 0.66

Toe clearance

	1 dpi	5 dpi	7 dpi	14 dpi	21 dpi	28 dpi	35 dpi	42 dpi
**PBS 6μl**	0 ± 0	0 ± 0	0 ± 0	0.5 ± 0.5	2.25 ± 0.63	2.25 ± 0.63	3 ± 0.41	3 ± 0.41
**(EIS)$_2$-RGD6 6μl**	0 ± 0	0 ± 0	0 ± 0	1.25 ± 0.48	2.5 ± 0.29	3.33 ± 0.67	3.67 ± 0.33	4 ± 0

Stepping

	1 dpi	5 dpi	7 dpi	14 dpi	21 dpi	28 dpi	35 dpi	42 dpi
**PBS 6μl**	0 ± 0	0 ± 0	0 ± 0	4.5 ± 0.96	6 ± 0	6 ± 0	6 ± 0	6 ± 0
**(EIS)$_2$-RGD6 6μl**	0 ± 0	0 ± 0	0 ± 0	5.75** ± 0.25	6 ± 0	6 ± 0	6 ± 0	6 ± 0

Tail position

	1 dpi	5 dpi	7 dpi	14 dpi	21 dpi	28 dpi	35 dpi	42 dpi
**PBS 6μl**	0 ± 0	0 ± 0	0 ± 0	0.5 ± 0.29	1 ± 0.41	1.25 ± 0.48	1.25 ± 0.25	1.25 ± 0.25
**(EIS)$_2$-RGD6 6μl**	0 ± 0	0 ± 0	0 ± 0	0.75 ± 0.25	1 ± 0	1 ± 0	1 ± 0	1.33 ± 0.33

Coordination

	1 dpi	5 dpi	7 dpi	14 dpi	21 dpi	28 dpi	35 dpi	42 dpi
**PBS 6μl**	0 ± 0	0 ± 0	0 ± 0	0 ± 0	0 ± 0	0.75 ± 0.25	1.5 ± 0.29	2 ± 0.41
**(EIS)$_2$-RGD6 6μl**	0 ± 0	0 ± 0	0 ± 0	0 ± 0	0.5 ± 0.29	1.33 ± 0.33	2 ± 0.58	2 ± 0.58

C. PBS 2 + 2 + 2µl vs. (EIS)$_2$-RGD6 2 + 2 + 2µl:

Paw positions

	1 dpi	5 dpi	7 dpi	14 dpi	21 dpi	28 dpi	35 dpi	42 dpi
**PBS 2 + 2 + 2µl**	0 ± 0	0 ± 0	0 ± 0	0.4 ± 0.4	0.8 ± 0.37	1.6 ± 0.68	1.4 ± 0.68	1.6 ± 0.6
**(EIS)$_2$-RGD6 2 + 2 + 2µl**	0 ± 0	0 ± 0	0 ± 0	1 ± 0	1 ± 0	1.75 ± 0.25	1.75 ± 0.25	2 ± 0

Toe clearance

	1 dpi	5 dpi	7 dpi	14 dpi	21 dpi	28 dpi	35 dpi	42 dpi
**PBS 2 + 2 + 2µl**	0 ± 0	0 ± 0	0 ± 0	0.8 ± 0.58	1.2 ± 0.8	1.8 ± 0.66	2.4 ± 0.4	3.2 ± 0.8
**(EIS)$_2$-RGD6 2 + 2 + 2µl**	0 ± 0	0 ± 0	0 ± 0	1.25 ± 0.48	1.5 ± 0.29	2 ± 0	2.5 ± 0.29	2.5 ± 0.29

Stepping

	1 dpi	5 dpi	7 dpi	14 dpi	21 dpi	28 dpi	35 dpi	42 dpi
**PBS 2 + 2 + 2µl**	0 ± 0	0 ± 0	0.5 ± 0.34	4 ± 2	5.6 ± 0.4	6 ± 0	6 ± 0	6 ± 0
**(EIS)$_2$-RGD6 2 + 2 + 2µl**	0 ± 0	0 ± 0	0 ± 0	5 ± 0.58	6 ± 0	6 ± 0	6 ± 0	6 ± 0

Tail position

	1 dpi	5 dpi	7 dpi	14 dpi	21 dpi	28 dpi	35 dpi	42 dpi
**PBS 2 + 2 + 2µl**	0 ± 0	0 ± 0	0 ± 0	0.6 ± 0.4	0.8 ± 0.2	1 ± 0.32	1.2 ± 0.37	1.2 ± 0.2
**(EIS)$_2$-RGD6 2 + 2 + 2µl**	0 ± 0	0 ± 0	0 ± 0	0 ± 0	0.5 ± 0.29	0.75 ± 0.25	1 ± 0.41	1.25 ± 0.25

Coordination

	1 dpi	5 dpi	7 dpi	14 dpi	21 dpi	28 dpi	35 dpi	42 dpi
**PBS 2 + 2 + 2µl**	0 ± 0	0 ± 0	0 ± 0	0 ± 0	0 ± 0	0.2 ± 0.2	0.8 ± 0.2	1.4 ± 0.24
**(EIS)$_2$-RGD6 2 + 2 + 2µl**	0 ± 0	0 ± 0	0 ± 0	0 ± 0	0.25 ± 0.25	1.25*** ± 0.48	1.5 ± 0.29	1.5 ± 0.29

D. PBS 3 + 6 + 3μl vs. (EIS)$_2$-RGD6 3 + 6 + 3μl:

Paw positions

	1 dpi	5 dpi	7 dpi	14 dpi	21 dpi	28 dpi	35 dpi	42 dpi
**PBS 3 + 6 + 3μl**	0 ± 0	0 ± 0	0 ± 0	0.2 ± 0.2	1 ± 0.32	1 ± 0.32	1.2 ± 0.2	1.6 ± 0.24
**(EIS)$_2$-RGD6 3 + 6 + 3μl**	0 ± 0	0 ± 0	0 ± 0	1.5** ± 0.64	1.25 ± 0.25	1.25 ± 0.25	1.67 ± 0.67	2.33 ± 0.33

Toe clearance

	1 dpi	5 dpi	7 dpi	14 dpi	21 dpi	28 dpi	35 dpi	42 dpi
**PBS 3 + 6 + 3μl**	0 ± 0	0 ± 0	0 ± 0	0.2 ± 0.2	0.6 ± 0.4	1.4 ± 0.24	1.6 ± 0.24	1.8 ± 0.37
**(EIS)$_2$-RGD6 3 + 6 + 3μl**	0 ± 0	0 ± 0	0 ± 0	1.5 ± 0.87	2.25* ± 0.85	2.75 ± 0.94	3.67** ± 0.67	3.67* ± 0.33

Stepping

	1 dpi	5 dpi	7 dpi	14 dpi	21 dpi	28 dpi	35 dpi	42 dpi
**PBS 3 + 6 + 3μl**	0 ± 0	0 ± 0	0 ± 0	4.8 ± 0.73	6 ± 0	6 ± 0	6 ± 0	6 ± 0
**(EIS)$_2$-RGD6 3 + 6 + 3μl**	0 ± 0	0 ± 0	0 ± 0	5.75*** ± 0.25	6 ± 0	6 ± 0	6 ± 0	6 ± 0

Tail position

	1 dpi	5 dpi	7 dpi	14 dpi	21 dpi	28 dpi	35 dpi	42 dpi
**PBS 3 + 6 + 3μl**	0 ± 0	0 ± 0	0 ± 0	0 ± 0	0.6 ± 0.24	0.6 ± 0.24	0.8 ± 0.37	0.8 ± 0.37
**(EIS)$_2$-RGD6 3 + 6 + 3μl**	0 ± 0	0 ± 0	0 ± 0	0.5 ± 0.29	0.75 ± 0.25	1 ± 0.41	0.68 ± 0.33	0.67 ± 0.33

Coordination

	1 dpi	5 dpi	7 dpi	14 dpi	21 dpi	28 dpi	35 dpi	42 dpi
**PBS 3 + 6 + 3μl**	0 ± 0	0 ± 0	0 ± 0	0 ± 0	0 ± 0	0.2 ± 0.2	0.8 ± 0.2	1 ± 0.32
**(EIS)$_2$-RGD6 3 + 6 + 3μl**	0 ± 0	0 ± 0	0 ± 0	0 ± 0	0.5 ± 0.29	1.5*** ± 0.28	2*** ± 0	2*** ± 0

E. PBS 12μl vs. $(EIS)_2$-RGD6 12μl:

Paw positions

	1 dpi	5 dpi	7 dpi	14 dpi	21 dpi	28 dpi	35 dpi	42 dpi
**PBS 12μl**	0 ± 0	0 ± 0	0 ± 0	0 ± 0	0.75 ± 0.75	0.5 ± 0.5	0.75 ± 0.48	0.5 ± 0.5
**$(EIS)_2$-RGD6 12μl**	0 ± 0	0 ± 0	0 ± 0	1.4 ± 0.63	2 ± 0.89	1.75 ± 0.87	1.67 ± 0.96	1.67 ± 0.96

Toe clearance

	1 dpi	5 dpi	7 dpi	14 dpi	21 dpi	28 dpi	35 dpi	42 dpi
**PBS 12μl**	0 ± 0	0 ± 0	0 ± 0	0 ± 0	0.5 ± 0.5	0.5 ± 0.5	0.75 ± 0.47	0.5 ± 0.5
**$(EIS)_2$-RGD6 12μl**	0 ± 0	0 ± 0	0 ± 0	1.2 ± 0.54	1.8 ± 0.8	3*** ± 1.5	2.67* ± 1.54	2.33* ± 1.35

Stepping

	1 dpi	5 dpi	7 dpi	14 dpi	21 dpi	28 dpi	35 dpi	42 dpi
**PBS 12μl**	0 ± 0	0 ± 0	0 ± 0	2.75 ± 1.25	2.75 ± 1.25	5 ± 1	5.75 ± 0.25	5 ± 1
**$(EIS)_2$-RGD6 12μl**	0 ± 0	0 ± 0	0 ± 0	4.8 ± 0.58	6** ± 0	6 ± 0	6 ± 0	6 ± 0

Tail position

	1 dpi	5 dpi	7 dpi	14 dpi	21 dpi	28 dpi	35 dpi	42 dpi
**PBS 12μl**	0 ± 0	0 ± 0	0 ± 0	0 ± 0	0 ± 0	0 ± 0	0.25 ± 0.25	0.75 ± 0.25
**$(EIS)_2$-RGD6 12μl**	0 ± 0	0 ± 0	0 ± 0	0 ± 0	0.8*** ± 0.36	1*** ± 0.5	1.33*** ± 0.77	1.33 ± 0.77

Coordination

	1 dpi	5 dpi	7 dpi	14 dpi	21 dpi	28 dpi	35 dpi	42 dpi
**PBS 12μl**	0 ± 0	0 ± 0	0 ± 0	0 ± 0	0 ± 0	0.25 ± 0.25	0.75 ± 0.25	0.75 ± 0.25
**$(EIS)_2$-RGD6 12μl**	0 ± 0	0 ± 0	0 ± 0	0.2 ± 0.09	0.8** ± 0.36	0.75 ± 0.375	1 ± 0.58	1 ± 0.58

F. Non injected (NI) group vs. groups injected with $(EIS)_2$-RGD6:

Paw positions

	1 dpi	5 dpi	7 dpi	14 dpi	21 dpi	28 dpi	35 dpi	42 dpi
**NI**	0 ± 0	0 ± 0	0 ± 0	1.25 ± 0.63	2.25 ± 0.85	2.5 ± 0.87	2.5 ± 0.65	2.25 ± 0.75
**$(EIS)_2$-RGD6 6µl**	0 ± 0	0 ± 0	0 ± 0	0.75 ± 0.5	1.5 ± 0.5	2.33 ± 0.33	2.67 ± 0.33	2.33 ± 0.66
**$(EIS)_2$-RGD6 2 + 2 + 2µl**	0 ± 0	0 ± 0	0 ± 0	1 ± 0	1 ± 0	1.75 ± 0.25	1.75 ± 0.25	2 ± 0
**$(EIS)_2$-RGD6 3 + 6 + 3µl**	0 ± 0	0 ± 0	0 ± 0	1.5 ± 0.64	1.25 ± 0.25	1.25 ± 0.25	1.67 ± 0.67	2.33 ± 0.33
**$(EIS)_2$-RGD6 12µl**	0 ± 0	0 ± 0	0 ± 0	1.4 ± 0.63	2 ± 0.89	1.75 ± 0.87	1.67 ± 0.96	1.67 ± 0.96

Toe clearance

	1 dpi	5 dpi	7 dpi	14 dpi	21 dpi	28 dpi	35 dpi	42 dpi
**NI**	0 ± 0	0 ± 0	0 ± 0	2 ± 0.71	4.25 ± 1.18	4.5 ± 0.5	4.25 ± 0.48	4.25 ± 0.48
**$(EIS)_2$-RGD6 6µl**	0 ± 0	0 ± 0	0 ± 0	1.25 ± 0.48	2.5 ± 0.29	3.33 ± 0.67	3.67 ± 0.33	4 ± 0
**$(EIS)_2$-RGD6 2 + 2 + 2µl**	0 ± 0	0 ± 0	0 ± 0	1.25 ± 0.48	1.5*** ± 0.29	2** ± 0	2.5 ± 0.29	2.5 ± 0.29
**$(EIS)_2$-RGD6 3 + 6 + 3µl**	0 ± 0	0 ± 0	0 ± 0	1.5 ± 0.87	2.25 ± 0.85	2.75 ± 0.94	3.67 ± 0.67	3.67 ± 0.33
**$(EIS)_2$-RGD6 12µl**	0 ± 0	0 ± 0	0 ± 0	1.2 ± 0.54	1.8** ± 0.8	3 ± 1.5	2.67 ± 1.54	2.33* ± 1.35

Stepping

	1 dpi	5 dpi	7 dpi	14 dpi	21 dpi	28 dpi	35 dpi	42 dpi
**NI**	0 ± 0	0 ± 0	2 ± 0.7	6 ± 0	6 ± 0	6 ± 0	6 ± 0	6 ± 0
**$(EIS)_2$-RGD6 6µl**	0 ± 0	0 ± 0	0*** ± 0	5.75 ± 0.25	6 ± 0	6 ± 0	6 ± 0	6 ± 0
**$(EIS)_2$-RGD6 2 + 2 + 2µl**	0 ± 0	0 ± 0	0*** ± 0	5* ± 0.58	6 ± 0	6 ± 0	6 ± 0	6 ± 0
**$(EIS)_2$-RGD6 3 + 6 + 3µl**	0 ± 0	0 ± 0	0*** ± 0	5.75 ± 0.25	6 ± 0	6 ± 0	6 ± 0	6 ± 0
**$(EIS)_2$-RGD6 12µl**	0 ± 0	0 ± 0	0*** ± 0	4.8** ± 0.58	6 ± 0	6 ± 0	6 ± 0	6 ± 0

Tail position

	1 dpi	5 dpi	7 dpi	14 dpi	21 dpi	28 dpi	35 dpi	42 dpi
**NI**	0 ± 0	0 ± 0	0 ± 0	0.25 ± 0.25	0.75 ± 0.25	1 ± 0.41	0.75 ± 0.25	0.75 ± 0.25
**$(EIS)_2$-RGD6 6µl**	0 ± 0	0 ± 0	0 ± 0	0.75 ± 0.25	1 ± 0	1 ± 0	1 ± 0	1.33 ± 0.33
**$(EIS)_2$-RGD6 2 + 2 + 2µl**	0 ± 0	0 ± 0	0 ± 0	0 ± 0	0.5 ± 0.29	0.75 ± 0.25	1 ± 0.41	1.25 ± 0.25
**$(EIS)_2$-RGD6 3 + 6 + 3µl**	0 ± 0	0 ± 0	0 ± 0	0.5 ± 0.29	0.75 ± 0.25	1 ± 0.41	0.68 ± 0.33	0.67 ± 0.33
**$(EIS)_2$-RGD6 12µl**	0 ± 0	0 ± 0	0 ± 0	0 ± 0	0.8 ± 0.36	1 ± 0.5	1.33 ± 0.77	1.33 ± 0.77

Coordination

	1 dpi	5 dpi	7 dpi	14 dpi	21 dpi	28 dpi	35 dpi	42 dpi
**NI**	0 ± 0	0 ± 0	0 ± 0	0.75 ± 0.48	1.75 ± 0.48	1.75 ± 0.48	2 ± 0.4	2.25 ± 0.48
**$(EIS)_2$-RGD6 6µl**	0 ± 0	0 ± 0	0 ± 0	0 ± 0	0.5 ± 0.29	1.33 ± 0.33	2 ± 0.58	2 ± 0.58
**$(EIS)_2$-RGD6 2 + 2 + 2µl**	0 ± 0	0 ± 0	0 ± 0	0 ± 0	0.25* ± 0.25	1.25 ± 0.48	1.5 ± 0.29	1.5 ± 0.29
**$(EIS)_2$-RGD6 3 + 6 + 3µl**	0 ± 0	0 ± 0	0 ± 0	0 ± 0	0.5* ± 0.29	1.5 ± 0.28	2 ± 0	2 ± 0
**$(EIS)_2$-RGD6 12µl**	0 ± 0	0 ± 0	0 ± 0	0.2 ± 0.09	0.8 ± 0.36	0.75 ± 0.375	1 ± 0.58	1* ± 0.58

Tables show the separate analysis of the individual BBB aspects assessed (paw positions, toe clearance, stepping, tail position, and coordination), at 1, 5, 7, 14, 28, and 42 days post-injury (dpi), in animals used to perform experiment $(EIS)_2$-RGD6 I. The following comparisons were performed: (A) Non-injected (NI) group vs. PBS-injected groups (PBS 6 µL, PBS 2 + 2 + 2 µL, PBS 3 + 6 + 3 µL, or PBS 12 µL) (*, $p < 0.05$; **, $p < 0.01$; and ***, $p < 0.001$ vs. NI group); (B) PBS 6 µL group vs. $(EIS)_2$-RGD6 6 µL group (**, $p < 0.01$ vs. PBS 6 µL group); (C) PBS 2 + 2 + 2 µL vs. $(EIS)_2$-RGD6 2 + 2 + 2 µL (***, $p < 0.001$ vs. PBS 2 + 2 + 2 µL); (D) PBS 3 + 6 + 3 µL vs. $(EIS)_2$-RGD6 3 + 6 + 3 µL (*, $p < 0.05$; **, $p < 0.01$; and ***, $p < 0.001$ vs. PBS 3 + 6 + 3 µL); (E), PBS 12 µL vs. $(EIS)_2$-RGD6 12 µL (*, $p < 0.05$; **, $p < 0.01$; and ***, $p < 0.001$ vs. PBS 12 µL), and (F) NI

group vs. $(EIS)_2$-RGD6-injected groups [$(EIS)_2$-RGD6 6 μL, $(EIS)_2$-RGD6 2 + 2 + 2 μL, $(EIS)_2$-RGD6 3 + 6 + 3 μL or $(EIS)_2$-RGD6 12 μL] (*, $p < 0.05$; **, $p < 0.01$; and ***, $p < 0.001$ vs. NI group). In all cases, the potential existence of statistically significant between-group differences was assessed by two-way ANOVA followed by Bonferroni post-hoc test. Please note that the combined analysis of these individual BBB aspects, in the form of BBB score and sub-score, can be found in Figures 1 and 2, respectively. In all cases, data are presented as the mean + SEM.
