# Supplementary material for: Silk-Elastin-like Polymers for Acute Intraparenchymal Treatment of the Traumatically Injured Spinal Cord: A First Systematic Experimental Approach
Source: Pharmaceutics. 2022 Dec 3;14(12):2713. doi: 10.3390/pharmaceutics14122713 (PMC9784492; doi:10.3390/pharmaceutics14122713)
Supplement: Supplementary file 1 [file pharmaceutics-14-02713-s001.zip › Table S2.pdf]

A. PBS (all) vs. $(EIS)_2$-RGD6 (all):

	Live	Death	Total
**PBS (all)**	18	2	20
**$(EIS)_2$-RGD6 (all)**	14	6	20
**Total**	20	20	40

B. PBS (3 + 6 + 3μl) vs. $(EIS)_2$-RGD6 (3 + 6 + 3μl):

	Live	Death	Total
**PBS (3 + 6 + 3μl)**	5	0	5
**$(EIS)_2$-RGD6 (3 + 6 + 3μl)**	3	3	6
**Total**	8	3	11

C. PBS (12μl) vs. $(EIS)_2$-RGD6 (12μl):

	Live	Death	Total
**PBS (12μl)**	4	0	4
**$(EIS)_2$-RGD6 (12μl)**	3	2	5
**Total**	7	2	9

D. PBS (2 + 2 + 2μl) vs. $(EIS)_2$-RGD6 (2 + 2 + 2μl):

	Live	Death	Total
**PBS (2 + 2 + 2μl)**	5	1	6
**$(EIS)_2$-RG6 (2 + 2 + 2μl)**	4	0	4
**Total**	9	1	10

E. PBS (6μl) vs. $(EIS)_2$-RGD6 (6μl):

	Live	Death	Total
**PBS (6μl)**	4	1	5
**$(EIS)_2$-RGD6 (6μl)**	4	1	5
**Total**	5	5	10

F. PBS (12 and 3 + 6 + 3μl) vs. $(EIS)_2$-RGD6 (12 and 3 + 6 + 3μl):

	Live	Death	Total
**PBS (12 and 3 + 6 + 3μl)**	9	0	9
**$(EIS)_2$-RGD6 (12 and 3 + 6 + 3μl)**	6	5*	11
**Total**	15	5	20

G. PBS (6 and 2 + 2 + 2μl) vs. $(EIS)_2$-RGD6 (6 and 2 + 2 + 2μl):

	Live	Death	Total
**PBS (6 and 2 + 2 + 2μl)**	9	2	11
**$(EIS)_2$-RGD6 (6 and 2 + 2 + 2μl)**	8	1	9
**Total**	17	3	20

Supplementary material including contingency tables corresponding to the analysis of mortality rate in animals used to perform experiment $(EIS)_2$-RGD6 I. The following comparisons were performed: (A) All PBS-injected animals (PBS 6 μL, PBS 2 + 2 + 2 μL, PBS 3 + 6 + 3 μL, and PBS 12 μL groups) vs. all $(EIS)_2$-RGD6-injected animals [$(EIS)_2$-RGD6 6 μL, $(EIS)_2$-RGD6 2 + 2 + 2 μL, $(EIS)_2$-RGD6 3 + 6 + 3 μL, and $(EIS)_2$-RGD6 12 μL]; (B) PBS 3 + 6 + 3 μL group vs. $(EIS)_2$-RGD6 3 + 6 + 3 μL group; (C) PBS 12 μL group vs. $(EIS)_2$-RGD6 12 μL group; (D) PBS 2 + 2 + 2 μL group vs. $(EIS)_2$-RGD6 2 + 2 + 2 μL group; (E) PBS 6 μL group vs. $(EIS)_2$-RGD6 6 μL group; (F) all animals injected with 12 μL of PBS (PBS 3 + 6 + 3 μL and PBS 12 μL groups) vs. all animals injected with 12 μL of $(EIS)_2$-RGD6 [$(EIS)_2$-RGD6 3 + 6 + 3 μL and $(EIS)_2$-RGD6 12 μL groups] (*, $p < 0.05$); and (G) all animals injected with 6 μL of PBS (PBS 2 + 2 + 2 μL and PBS 6 μL groups) vs. all animals injected with 6 μL of $(EIS)_2$-RGD6 [$(EIS)_2$-RGD6 2 + 2 + 2 μL and $(EIS)_2$-RGD6 6 μL groups]. In A–G, the potential existence of statistically significant between-group differences was assessed by Fisher's exact test. Please note that a graphical representation of the mortality analysis can be found in Figure 8.
