# Supplementary material for: Silk-Elastin-like Polymers for Acute Intraparenchymal Treatment of the Traumatically Injured Spinal Cord: A First Systematic Experimental Approach
Source: Pharmaceutics. 2022 Dec 3;14(12):2713. doi: 10.3390/pharmaceutics14122713 (PMC9784492; doi:10.3390/pharmaceutics14122713)
Supplement: Supplementary file 1 [file pharmaceutics-14-02713-s001.zip › Table S3.pdf]

Paw positions

	1 dpi	5 dpi	7 dpi	14 dpi	21 dpi	28 dpi	35 dpi	42 dpi
**PBS 6µl**	0 ± 0	0 ± 0	0 ± 0	0.12 ± 0.12	0.12 ± 0.12	0.62 ± 0.42	0.43 ± 0.43	1.33 ± 0.42
**(EIS)2-RGD6 6µl**	0 ± 0	0 ± 0	0 ± 0	1.08 ± 0.33	1.91*** ± 0.44	2.1** ± 0.48	1.7* ± 0.39	2.1 ± 0.38

Toe clearance

	1 dpi	5 dpi	7 dpi	14 dpi	21 dpi	28 dpi	35 dpi	42 dpi
**PBS 6µl**	0 ± 0	0 ± 0	0 ± 0	0.5 ± 0.5	1.25 ± 0.59	1.62 ± 0.71	2.14 ± 0.83	2.83 ± 0.65
**(EIS)2-RGD6 6µl**	0 ± 0	0 ± 0	0 ± 0	0.08 ± 0.08	1.36 ± 0.56	2.3 ± 0.63	2.7 ± 0.76	2.6 ± 0.73

Stepping

	1 dpi	5 dpi	7 dpi	14 dpi	21 dpi	28 dpi	35 dpi	42 dpi
**PBS 6µl**	0 ± 0	0 ± 0	0 ± 0	2.75 ± 0.65	6 ± 0	6 ± 0	6 ± 0	6 ± 0
**(EIS)2-RGD6 6µl**	0 ± 0	0 ± 0	0.11 ± 0.1	4.5*** ± 0.7	6 ± 0	6 ± 0	6 ± 0	6 ± 0

Tail position

	1 dpi	5 dpi	7 dpi	14 dpi	21 dpi	28 dpi	35 dpi	42 dpi
**PBS 6µl**	0 ± 0	0 ± 0	0 ± 0	0.375 ± 0.26	0.75 ± 0.25	0.75 ± 0.16	0.86 ± 0.26	1.5 ± 0.22
**(EIS)2-RGD6 6µl**	0 ± 0	0 ± 0	0 ± 0	0.416 ± 0.15	0.45 ± 0.16	0.5 ± 0.17	0.5 ± 0.22	0.6* ± 0.27

Coordination

	1 dpi	5 dpi	7 dpi	14 dpi	21 dpi	28 dpi	35 dpi	42 dpi
**PBS 6µl**	0 ± 0	0 ± 0	0 ± 0	0.12 ± 0.12	0.625 ± 0.32	1.37 ± 0.18	1.29 ± 0.18	1.83 ± 0.31
**(EIS)2-RGD6 6µl**	0 ± 0	0 ± 0	0 ± 0	0 ± 0	0.81 ± 0.23	1.4 ± 0.22	1.7 ± 0.3	1.9 ± 0.31

Supplementary tables show the separate analysis of the individual BBB aspects assessed (paw positions, toe clearance, stepping, tail position, and coordination), at 1, 5, 7, 14, 28, and 42 days post-injury (dpi), in animals used to perform experiment $(EIS)_2$-RGD6 III. In all cases, the potential existence of statistically significant differences between PBS 6 µL and $(EIS)_2$-RGD6 6 µL groups was assessed by two-way ANOVA followed by Bonferroni post-hoc test (*, $p < 0.05$; **, $p < 0.01$; and ***, $p < 0.001$ vs. PBS 6 µL group). Please note that the combined analysis of these individual BBB aspects, in the form of BBB score and sub-score, are shown in Figure 15A,B, respectively. In all cases, data are presented as the mean + SEM.
